# Identification of crucial inflammaging related risk factors in multiple sclerosis

**DOI:** 10.3389/fnmol.2024.1398665

**Published:** 2024-05-21

**Authors:** Mengchu Xu, Huize Wang, Siwei Ren, Bing Wang, Wenyan Yang, Ling Lv, Xianzheng Sha, Wenya Li, Yin Wang

**Affiliations:** ^1^Department of Biomedical Engineering, School of Intelligent Sciences, China Medical University, Shenyang, Liaoning, China; ^2^Department of Nursing, First Affiliated Hospital of China Medical University, Shenyang, Liaoning, China; ^3^Department of Thorax, The First Hospital of China Medical University, Shenyang, Liaoning, China; ^4^Tumor Etiology and Screening Department of Cancer Institute and General Surgery, The First Affiliated Hospital of China Medical University, Shenyang, Liaoning, China

**Keywords:** inflammaging, multiple sclerosis, machine learning, network analysis, pan-cancer analysis

## Abstract

**Background:**

Multiple sclerosis (MS) is an immune-mediated disease characterized by inflammatory demyelinating lesions in the central nervous system. Studies have shown that the inflammation is vital to both the onset and progression of MS, where aging plays a key role in it. However, the potential mechanisms on how aging-related inflammation (inflammaging) promotes MS have not been fully understood. Therefore, there is an urgent need to integrate the underlying mechanisms between inflammaging and MS, where meaningful prediction models are needed.

**Methods:**

First, both aging and disease models were developed using machine learning methods, respectively. Then, an integrated inflammaging model was used to identify relative risk factors, by identifying essential “aging-inflammation-disease” triples. Finally, a series of bioinformatics analyses (including network analysis, enrichment analysis, sensitivity analysis, and pan-cancer analysis) were further used to explore the potential mechanisms between inflammaging and MS.

**Results:**

A series of risk factors were identified, such as the protein homeostasis, cellular homeostasis, neurodevelopment and energy metabolism. The inflammaging indices were further validated in different cancer types. Therefore, various risk factors were integrated, and even both the theories of inflammaging and immunosenescence were further confirmed.

**Conclusion:**

In conclusion, our study systematically investigated the potential relationships between inflammaging and MS through a series of computational approaches, and could present a novel thought for other aging-related diseases.

## 1 Introduction

Multiple sclerosis (MS) is an immune-mediated disease characterized by inflammatory demyelinating lesions in the central nervous system (CNS). MS is one of the major causes of disability (GBD 2016 Neurology Collaborators, 2019), leading to a heavy burden on families and society (Wang et al., 2023). It has been estimated that the number of people with MS increased to 2.8 million globally in 2020, 30% greater than that in 2013 (Walton et al., 2020). Therefore, it is imperative to study the underlying risk factors of MS.

There is substantial (i.e., epidemiological, pathological and clinical) evidence indicating that chronological age is as the factor mostly vital to MS (Graves et al., 2023), and even the development of MS is closely related to aging (Graves et al., 2023). For example, telomere abrasion is associated with disability and brain atrophy in MS patients (Krysko et al., 2019), and reproductive aging might also affect MS progression (Graves et al., 2018). Moreover, aging microglia create a chronic inflammatory microenvironment declining the normal function of remyelination (Neumann et al., 2019), and aging astrocytes are vital to impair synaptic plasticity and disturb the neuronal metabolic homeostasis (Correale and Farez, 2015; Oost et al., 2018). In short, there is growing evidence that aging promotes the development of MS.

MS is a chronic inflammatory disease closely relate to the aging process (where aging-related inflammation is often defined as inflammaging) (Xia et al., 2016; Cantuti-Castelvetri et al., 2022). The main pathological hallmark of MS was demyelinating plaque, which was also accompanied by chronic inflammation (Howe et al., 2007; Lemus et al., 2018). It has been reported that aging promoted neuroinflammation in MS and even led to a diminished ability of microglia responding to axonal deficits (Mestre et al., 2021). Moreover, senescent microglia were characterized by reduced migration and phagocytosis abilities, indicating that they were less efficient in removing myelin debris from damaged neurons in MS (Neumann et al., 2009). Furthermore, ongoing neuroinflammation was associated with neuronal death, which was vital to injure the neuronal health (Simkins et al., 2021). During inflammatory CNS episodes, several types of neurotoxic oxidation products were synthetized, thus leading to increased energy demands (Mahad et al., 2009; Haider et al., 2011). In conclusion, it could be speculated that the aging-related inflammation (inflammaging) was as one of the major risk factors in MS that needed to be explored more systematically.

Fortunately, with the development of artificial intelligence, many of the researches on MS utilized machine learning (ML), which allowed for the diagnosis and prognosis using real datasets (Aslam et al., 2022). In addition, ML techniques offered new insights in the diagnosis, characterization and prediction of disease progression (Jasperse and Barkhof, 2023). Several studies have shown that ML can recognize key markers associated with inflammation and aging (Mezzaroba et al., 2020; Zhou et al., 2023). Meanwhile, there was an urgent need to integrate key biomarkers and biological information (e.g., by mendelian randomization, MR) (Yuan et al., 2021; Li C. et al., 2022). In addition, gene co-expression network analysis could also identify highly correlated gene clusters and explore their potential molecular mechanisms in MS (Creanza et al., 2016; Gu et al., 2022). However, despite a large number of studies explaining the risk factors in MS, potential mechanisms of in MS based on inflammaging were still unclear and thus needed to be further explored at the system level.

**Figure 1:**
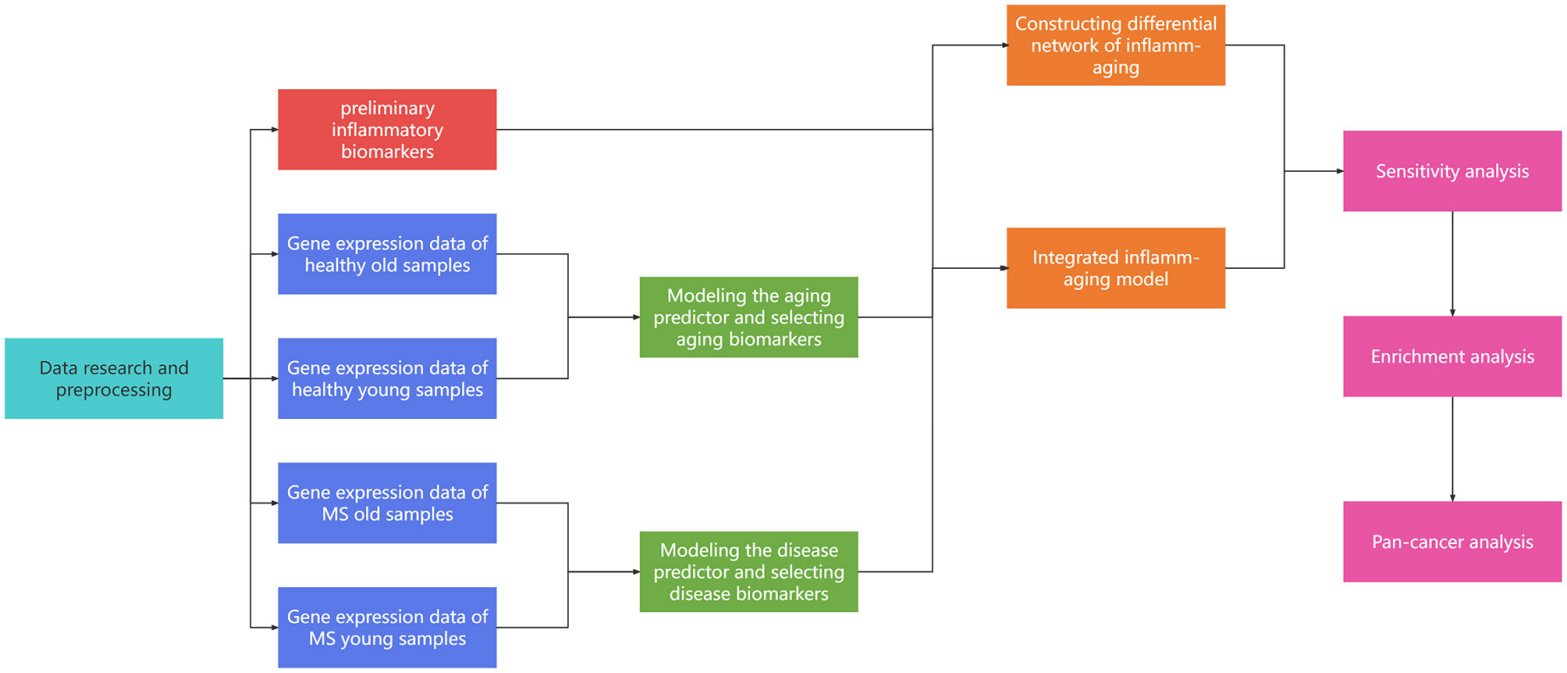
The workflow of our study.

To further explore the potential mechanisms involved in aging, inflammation and MS, a series of computational methods were integrated in this work (Figure 1): (1) Machine learning was used to identify aging and disease (MS) markers, respectively; (2) An integrated inflammaging model was used to explore the key relationship between inflammaging and MS, by identifying essential “aging-inflammation-disease” triples; (3) Network analysis, sensitivity analysis and enrichment analysis were used to study potential risk factors for multiple sclerosis; (4) Pan-cancer analysis was used to further validate relative biological functions in cancers based on “aging-inflammation-disease” triples. Ultimately, a series of underlying mechanisms of MS (i.e., protein homeostasis, cellular homeostasis, neurodevelopment, and energy metabolism) were integrated, which also provided key indicators for cancer. These results could also present a novel thought for other aging-relative experimental validations.

## 2 Results

### 2.1 Modeling prediction models and identifying relative biomarkers

**Figure 2:**
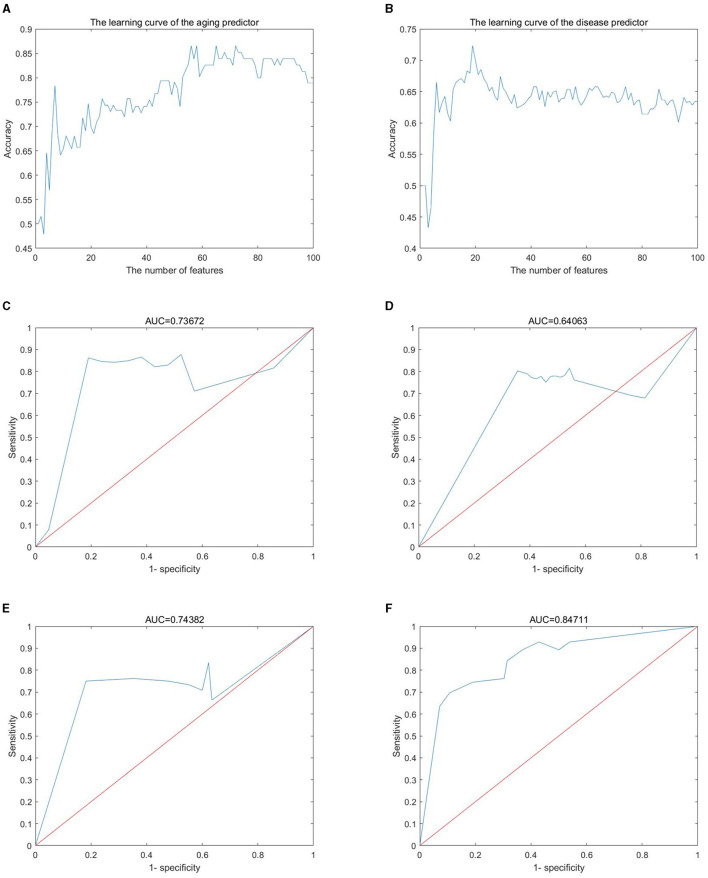
Machine learning results. **(A, B)** Learning curve for the training dataset; **(C, D)** sensitivity and specificity (similar to receiver operating characteristic) curves; **(E, F)** the ROC curve for the test dataset; **(A, C, E)** the aging model; **(B, D, F)** the disease (MS) model.

Gene expression profiles were obtained from the GEO database, including 445 samples (Supplementary Table S1) and 16,275 genes (Supplementary Table S2). These genes were ranked by the ReliefF algorithm, and then the aging predictor and disease predictor were modeled using the k-nearest neighbors (kNN; k = 9 with the correlation distance) algorithm and optimized by 10-fold cross-validation. The learning curves for the aging and disease models in the training dataset were shown in Figures 2A, B, where the models with the highest accuracy were selected (Table 1), including 70 aging markers and 19 disease markers (Supplementary Tables S3, S4). As a result, the accuracies of the aging model and the disease model in the test set were 0.8390 and 0.7233, respectively (Table 1). Furthermore, the areas under the curve (AUCs) for the aging and disease models in the test were 0.73672 and 0.64063 (Figures 2C, D), by summarizing the specificity (the accuracy for the normal old samples in the aging model, or for the MS samples in the disease model) and the sensitivity (the accuracy for the normal young samples in the aging model, or for the control samples in the disease model) based on the ReliefF ranking results (i.e., the first one gene expression, the first two genes, or the first three genes,..., the first 100 genes), respectively. In addition, the AUC of the ROC curves were 0.74382 and 0.84711 based on the aging and disease score, respectively (Figures 2E, F). Consequently, these results indicated that our predictors were with enough accuracies in both aging and disease models.

**Table 1:** The accuracy of the aging predictor model and disease predictor model.

	**The accuracy of training datasets**	**The accuracy of test datasets**	**Markers used for classification**
The aging predictor	0.8744	0.8390	70
The disease predictor	0.6853	0.7233	19

Both aging and disease markers indicated important biological functions. For example, TSPAN6 (tetraspanin 6, ReliefF weight was 0.135) was the top aging marker. It had been reported that TSPAN6 was as a novel regulator of APP-CTF protein homeostasis that prevented APP-CTF degradation from the impairment in autolysosomal pathway (Guix et al., 2017). In addition, TSPAN6 was identified as a regulator of synaptic transmission and plasticity mechanisms, playing a key role in synaptic development and AMPAR transport (Salas et al., 2017; Becic et al., 2022). POM121L9P (POM121 transmembrane nucleoporin like 9 as a pseudogene, ReliefF weight 0.051) was the top disease marker. FNDC4 (Fibronectin type III structural domain-containing protein 4, ReliefF weight was 0.050) was the second top disease marker, and it have been shown to induce the AMP-activated protein kinase (AMPK) phosphorylation and heme oxygenase-1 (HO-1) expression in adipocytes, which in turn suppressed inflammation and endoplasmic reticulum stress (Lee et al., 2018). In short, these results indicated potential mechanisms (i.e., neurodevelopment and energy metabolism) between aging and MS.

### 2.2 Identifying essential relationships in MS by the integrated inflammaging model

An integrated inflammaging model was developed to explore the important relationships among aging, inflammation and MS (shown in Section 5.3). Table 2 (Chen and D'Mello, 2010; Wan, 2014; Maridas et al., 2017; Jatczak-Pawlik et al., 2020; Tong et al., 2020; Black et al., 2021; Correale, 2021; Fadul et al., 2021; Sehgal et al., 2021; Atiyah et al., 2023) demonstrated the top ten aging, inflammatory and disease markers, respectively. For example, BMP8A (bone morphogenetic protein 8a), the top aging marker, can achieve anti-adiposity by promoting fatty acid oxidation and inhibiting adipocyte differentiation (Zhong et al., 2023). Here, FNDC4 was also identified as the top disease marker in triples. IDO1 (indoleamine 2,3-dioxygenase 1) was the top inflammatory marker as a key determinant enzyme in the metabolism of L-tryptophan (Trp), shifting the process from serotonin production to kynurenine production (Correale, 2021). The roles of the kynurenine pathway included endogenous regulation of neuronal excitability, initiation of immune tolerance and synthesis of nicotinamide adenine dinucleotide (NAD), where NAD+ being a key molecule in a variety of biochemical processes (Mbongue et al., 2015; Zhong et al., 2023). In summary, the integrated inflammaging model revealed important relationships among aging, inflammation and MS.

**Table 2:** The top ten aging, inflammatory, and disease markers from the integrated inflammaging model.

**Aging marker**	**Times**	**Disease marker**	**Times**	**Inflammatory marker**	**Times**	**Contents of reference**	**Experimental method**	**References**
BMP8A	48	POM121L9P	127	IDO1	51	IDO1 had a role in regulating neuronal excitability.	IDO1 gene-deficient mouse model	Correale, 2021
TMPRSS13	29	FNDC4	70	SLC18A2	25	SLC18A2 had a neuroprotective effect.	Human embryonic kidney cell culture and 96-well plate screening assay	Black et al., 2021
KHDRBS3	27	DCHS2	56	CD40LG	23	Blocking CD40LG might be an effective treatment for patients with MS.	Humanized (αCD40Ltoralizumab) IV infusion	Fadul et al., 2021
FAT1	23	SOBP	53	CAMK1D	22	CAMK1D caused increased protein expression levels and altered regulation of glucose processing.	Protein resonance assignment	Tong et al., 2020
FRAS1	22	CRYGB	45	IGFBP4	19	IGFBP4 might be a key regulator of adipose tissue development and maintenance.	Igfbp4 knockout mouse model	Maridas et al., 2017
CTNND2	20	CR2	34	PNMA1	19	PNMA1 could promote neuronal apoptosis.	Western blot and co-immunoprecipitation analysis	Chen and D'Mello, 2010
COL21A1	20	ADAM28	30	GATA3	14	GATA3 regulated T-cell development, proliferation and maintenance.	cDNA representational difference analysis	Wan, 2014
CXADR	19	PCDH9	27	TLR10	13	TLR10 was recognized as a novel inhibitor of the inflammatory responses, and was downregulation in serum of MS.	Enzyme-linked immunosorbent assay	Atiyah et al., 2023
CAP2	19	RASGRP3	26	CCR6	13	CCR6 on regulatory T cells might be a potential target for therapeutic intervention in MS.	Flow cytometry	Jatczak-Pawlik et al., 2020
AMOT	17	LOC647070	24	CSF1	13	CSF1 controlled the differentiation, survival, proliferation and renewal of monocytes and macrophages.	Injection of recombinant human CSF1 into a mouse model	Sehgal et al., 2021

### 2.3 Sensitivity analysis revealed crucial relationships between inflammaging and MS

The Markov Chain Monte Carlo (MCMC) method was used to assess the sensitivity indices between inflammaging and MS. As a result, the 35 sensitive triples (by calculating the absolute difference frequency) were shown in Table 3 (Sarasin-Filipowicz et al., 2009; Chen and D'Mello, 2010; Bergbold and Lemberg, 2013; Liu et al., 2013; Malhotra et al., 2013; Wan, 2014; Charbit et al., 2015; Fusco et al., 2015; An et al., 2017; Arentsen et al., 2017; Maridas et al., 2017; Mathur et al., 2017; Xiao et al., 2019; Immler et al., 2020; Sato et al., 2020; Tong et al., 2020; Buhelt et al., 2021; Correale, 2021; Fadul et al., 2021; Ma et al., 2021; Peng et al., 2021; Bogacka et al., 2022; Franceschi et al., 2022; Hjæresen et al., 2022; Khurana and Goswami, 2022; Liu S. et al., 2022; Schebb et al., 2022; Watanabe et al., 2022; Saeidi et al., 2023). For example, the sensitive triple with maximum difference was “TMPRSS13-USP18-DCHS2” (the absolute difference value was 0.270469). TMPRSS13 (transmembrane serine protease 13) had an essential role in its zymogen autoactivation, proteolytic activity to ward the protein substrate prostasin and phosphorylation (Martin et al., 2021). USP18 (Ubiquitin-specific peptidase 18) was a deubiquitinating enzyme, and as a negative regulator of type I IFN (IFN-α and IFN-β) signaling pathway, where IFN-β was effective for treating MS (Sarasin-Filipowicz et al., 2009; Malhotra et al., 2013; Charbit et al., 2015). In short, USP18 was vital to MS pathogenesis (Malhotra et al., 2013). DCHS2 (dachsous cadherin-related 2) has been implicated in the regulations of planar cell polarity and cell movement (such as convergence-extension and cell migration) (Lodge et al., 2020).

**Table 3:** Absolute difference frequencies for 35 triples in the sensitivity analysis.

**Aging marker**	**Inflammatory marker**	**Disease marker**	**Absolute difference frequencies**	**Contents of reference**	**Experimental method**	**References**
TMPRSS13	USP18	DCHS2	0.270469	USP18 was a deubiquitinating enzyme, related to IFN-β that can treat MS.	Real-time PCR	Sarasin-Filipowicz et al., 2009; Malhotra et al., 2013; Vécsei et al., 2013; Charbit et al., 2015
TMPRSS13	USP18	POM121L9P	0.250288	USP18 was a deubiquitinating enzyme, related to IFN-β that can treat MS.	Real-time PCR	Sarasin-Filipowicz et al., 2009; Malhotra et al., 2013; Vécsei et al., 2013; Charbit et al., 2015
TMPRSS13	PLA2G2D	DCHS2	0.246574	PLA2G2D increased energy expenditure and thermogenesis by facilitating adipocyte browning.	Global and macrophage-specific Pla2g2d deletion mouse models	Sato et al., 2020
TMPRSS13	CUEDC2	DCHS2	0.230594	CUEDC2 was involved in the cell cycle regulation and inflammation.	Real-time PCR and methylation-specific polymerase chain reaction	Xiao et al., 2019
TMPRSS13	MMP8	POM121L9P	0.223794	MMP8 could initiate the first step of collagen degradation and was associated with the initial inflammatory stages of wound repair.	DNA purification	An et al., 2017
TMPRSS13	XCL1	POM121L9P	0.206697	XCL1 might contribute to the migration of autoreactive T cells to the CNS and played a key role in the pathogenesis and development of MS.	Enzyme-linked immunosorbent assay (ELISA)	Saeidi et al., 2023
TMPRSS13	CAMK2N1	POM121L9P	0.195029	CAMK2N1 was involved in the negative regulation of cell population proliferation.	qRT-PCR and immunoblotting assays	Peng et al., 2021
TMPRSS13	SHPK	DCHS2	0.181103	SHPK catalyzed the phosphorylation of sedoheptulose in the non-oxidative arm of the pentose phosphate pathway.	Immunofluorescence and WST1 assay	Franceschi et al., 2022
TMPRSS13	IKBKG	POM121L9P	0.176078	IKBKG was involved in a variety of physiological and cellular processes, such as immunity, inflammation, cell proliferation, and survival.	Mutation sequence analysis	Fusco et al., 2015
BMP8A	CCR4	POM121L9P	0.126684	CCR4 was important in the pathogenesis of MS.	CCR4 knockout mouse model	Bogacka et al., 2022
TMPRSS13	USP18	CRYGB	0.126354	USP18 was a deubiquitinating enzyme, related to IFN-β that can treat MS.	Real-time PCR	Sarasin-Filipowicz et al., 2009; Malhotra et al., 2013; Charbit et al., 2015
CRYBG3	USP18	POM121L9P	0.125618	USP18 was a deubiquitinating enzyme, related to IFN-β that can treat MS.	Real-time PCR	Sarasin-Filipowicz et al., 2009; Malhotra et al., 2013; Charbit et al., 2015
FRAS1	IDO1	ADAM28	0.112627	IDO1 had a role in regulating neuronal excitability.	IDO1 gene-deficient mouse model	Correale, 2021
CAP2	IDO1	ARHGAP17	0.111331	IDO1 had a role in regulating neuronal excitability.	IDO1 gene-deficient mouse model	Correale, 2021
ATP10D	IL2RA	CRYGB	0.108406	IL2RA was involved in the pathogenesis of MS.	Flow cytometry analysis	Buhelt et al., 2021
CAP2	RHBDD3	POM121L9P	0.106237	RHBDD3 was a negative regulator of natural killer cell activation and positive regulator of protein catabolic process.	Flow cytometry, coimmunoprecipitation and western blot	Bergbold and Lemberg, 2013; Liu et al., 2013
CRHBP	CD40LG	FNDC4	0.104379	Blocking CD40LG might be an effective treatment for patients with MS.	Humanized αCD40L (toralizumab) IV infusion	Fadul et al., 2021
SGCD	UMOD	SOBP	0.104191	UMOD underwent proteolytic cleavage to pUMOD, which was the most abundant urinary protein in healthy individuals.	Immunofluorescence and transendothelial electrical resistance measurements	Immler et al., 2020
CAP2	GATA3	POM121L9P	0.103396	GATA3 regulated T-cell development, proliferation and maintenance.	cDNA representational difference analysis	Wan, 2014
BCMO1	XCL1	PCDH9	0.101988	XCL1 might contribute to the migration of autoreactive T cells to the CNS and played a key role in the pathogenesis and development of MS.	ELISA	Saeidi et al., 2023
BMP8A	SMPDL3B	POM121L9P	0.099676	SMPDL3B was involved in the negative regulation of toll-like receptor signaling pathway.	qRT-PCR analyses and western blot	Watanabe et al., 2022
FLJ11292	ALOX15	POM121L9P	0.08205	ALOX15 produced a variety of bioactive lipid mediators and played a role in the resolution of inflammation.	ELISA and LC-MS analysis of SPMs	Schebb et al., 2022
VPS13C	CAMK1D	DCHS2	0.075201	CAMK1D caused increased protein expression levels and altered regulation of glucose processing.	Protein resonance assignment	Tong et al., 2020
MPPED2	IDO1	FNDC4	0.074466	IDO1 had a role in regulating neuronal excitability.	IDO1 gene-deficient mouse model	Correale, 2021
POLR2H	IGFBP4	CRYGB	0.063038	IGFBP4 might be a key regulator of adipose tissue development and maintenance.	Igfbp4 knockout mouse model	Maridas et al., 2017
ATP10D	NCR3	FNDC4	0.056491			
TMPRSS13	IRF3	CRYGB	0.051158	IRF3 could inhibit STING pathway, which was a regulator of microglial reactivity and neuroinflammation.	qRT-PCR, siRNA knockdown and flow cytometry	Mathur et al., 2017
BMP8A	FABP4	POM121L9P	0.051109	FABP4 was responsible for promoting lipid storage, distribution, transportation, decomposition and metabolism.	Fluorescence in situ hybridization and somatic cell hybridization	Liu S. et al., 2022
FRAS1	TNFSF4	CR2	0.045746			
MPPED2	ACE	SOBP	0.030306	ACE was involved in regulating blood pressure.	Chromatographic and electrophoretic techniques	Khurana and Goswami, 2022
ELL2	PGLYRP2	ADAM28	0.027812	PGLYRP2 affected the development of neurons.	PGN detection assay, qPCR and western blot	Arentsen et al., 2017
CACNA2D2	ITIH4	POM121L9P	0.021254	ITIH4 played a role in inflammation and infection response.	Western blot and qRT-PCR	Ma et al., 2021
FLJ11292	MIF	POM121L9P	0.021218	MIF mediated neuroprotective effects by suppressing inflammatory responses, and inhibiting apoptosis.	ELISA	Hjæresen et al., 2022
COL21A1	IDO1	ADAM28	0.011991	IDO1 had a role in regulating neuronal excitability.	IDO1 gene-deficient mouse model	Correale, 2021
CRHBP	PNMA1	FNDC4	0.001599	PNMA1 could promote neuronal apoptosis.	Western blot and coimmunoprecipitation analysis	Chen and D'Mello, 2010

The top sensitive (with occurring times) aging, inflammatory and disease markers were also shown in Table 4 (Sarasin-Filipowicz et al., 2009; Chen and D'Mello, 2010; Malhotra et al., 2013; Charbit et al., 2015; An et al., 2017; Arentsen et al., 2017; Lee et al., 2018; Xiao et al., 2019; Sato et al., 2020; Peng et al., 2021; Franceschi et al., 2022; Saeidi et al., 2023). For example, the top aging and inflammatory markers were also TMPRSS13 and USP18, respectively. POM121L9P was a pseudogene and the second disease marker was DCHS2. In summary, the sensitive analysis indicated that protein homeostasis and cellular homeostasis played important roles in the development of MS.

**Table 4:** Sensitivity analysis of the top 10 genes related to aging, disease, and inflammation.

**Aging marker**	**Times**	**Disease marker**	**Times**	**Inflammatory marker**	**Times**	**Contents of reference**	**Experimental method**	**References**
TMPRSS13	11	POM121L9P	14	USP18	4	USP18 was a deubiquitinating enzyme, related to IFN-β that can treat MS	Real-time PCR	Sarasin-Filipowicz et al., 2009; Malhotra et al., 2013; Charbit et al., 2015
CAP2	3	DCHS2	5	IDO1	4	IDO1 was involved in the catabolism of amino acids.	IDO1 gene-deficient mouse model	Correale, 2021
BMP8A	3	CRYGB	4	XCL1	2	XCL1 might contribute to the migration of autoreactive T cells to the CNS and played a key role in the pathogenesis and development of MS.	ELISA	Saeidi et al., 2023
MPPED2	2	FNDC4	4	CUEDC2	1	CUEDC2 was involved in the cell cycle regulation and inflammation.	Real-time PCR and methylation-specific polymerase chain reaction	Xiao et al., 2019
CRHBP	2	ADAM28	3	CAMK2N1	1	CAMK2N1 inhibited HCC and colorectal carcinoma, and modulated obesity by affecting many metabolic syndrome features.	qRT-PCR and immunoblotting assays	Peng et al., 2021
ATP10D	2	SOBP	2	PLA2G2D	1	PLA2G2D increased energy expenditure and thermogenesis by facilitating adipocyte browning, thereby ameliorating diet-induced obesity and WAT inflammation.	Global and macrophage-specific Pla2g2d deletion mouse models	Sato et al., 2020
FRAS1	2	ARHGAP17	1	SHPK	1	SHPK catalyzed the phosphorylation of sedoheptulose in the non-oxidative arm of the pentose phosphate pathway and was associated with energy metabolism.	Immunofluorescence and WST1 assay	Franceschi et al., 2022
FLJ11292	2	PCDH9	1	MMP8	1	MMP8 could initiate the first step of collagen degradation and was associated with initial inflammatory stages of wound repair.	DNA purification	An et al., 2017
CRYBG3	1	CR2	1	PGLYRP2	1	PGLYRP2 affected the development of neurons.	PGN detection assay, qPCR and western blot	Arentsen et al., 2017
ELL2	1			PNMA1	1	PNMA1 could promote neuronal apoptosis.	Western blot and coimmunoprecipitation analysis	Chen and D'Mello, 2010

### 2.4 Underlying inflammaging mechanisms by enrichment analysis

To further explore potential underlying mechanisms between inflammaging and MS, each shortest path between inflammatory and disease markers was obtained based on the Dijkstra algorithm, then the enrichment analysis was performed based on Kyoto Encyclopedia of Genes and Genomes (KEGG) pathway and Biological Process (BP) terms in Gene Ontology (GO). Because the starting point in each shortest path was a inflammatory marker, the enriched functions was analyzed by deleting the starting point, or analyzed other enriched functions excluding inflammation related function if containing the starting point.

**Figure 3:**
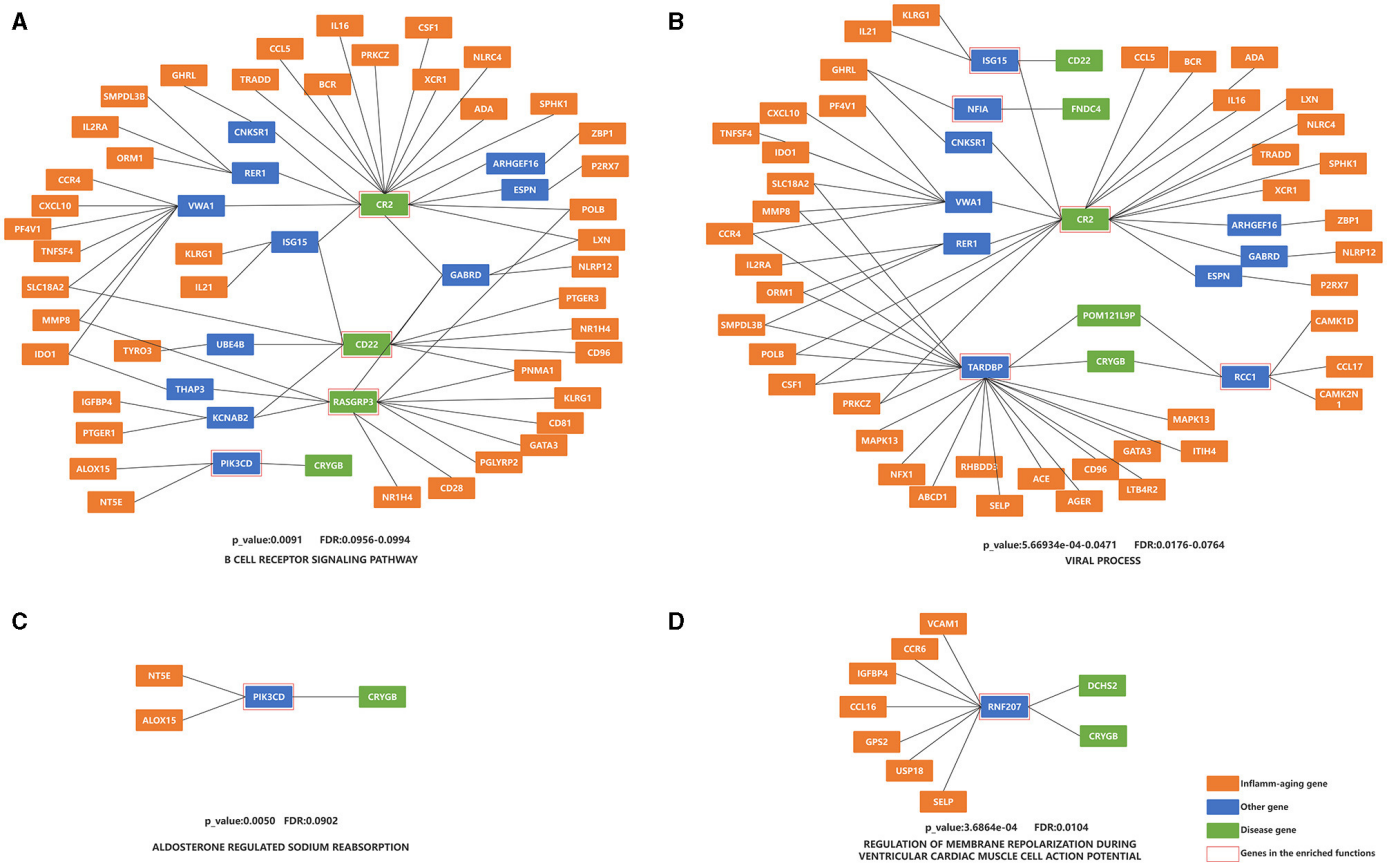
Enrichment analysis of the shortest paths of KEGG and BP, after combining overlap shortest paths. **(A)** KEGG with the most shortest enriched paths; **(B)** BP with the most shortest enriched paths; **(C)** KEGG with the minimum FDR; **(D)** BP with the minimum FDR. The orange nodes represent the inflammaging markers, the blue nodes represent the genes connecting inflammaging markers and disease markers, the green nodes represent the disease markers, and the genes in the red square frames coincide with those genes in the enriched functions.

The top ten KEGG pathways were shown in Table 5 (Lasky, 1991; Reichardt, 2006; Fujita et al., 2009; O'Callaghan et al., 2017; Conway, 2018; Gao et al., 2018; Kotelnikova et al., 2019; Plantone et al., 2019; Ten Bosch et al., 2021; Bahadoram et al., 2022; Bohmwald et al., 2022; Sen et al., 2022; Wu and Zhou, 2022; Chen et al., 2023; Liu et al., 2023; Touil et al., 2023; Guerra-Espinosa et al., 2024) and Figure 3 and Supplementary Figure S1 (without the starting point). For example, the KEGG pathway that was most enriched shortest paths was “B CELL RECEPTOR SIGNALING PATHWAY” (enriched 50 shortest paths, Figure 3A and Supplementary Figure S1A). BCR was critical for B cells to properly elicit an immune response (Tanaka and Baba, 2020). The KEGG pathway with minimum FDR was “ALDOSTERONE REGULATED SODIUM REABSORPTION” (FDR = 0.0902, Figure 3C and Supplementary Figure S1C), where sodium reabsorption occurred in the kidney (Franken et al., 2021). Sodium accumulation might play a critical role in both inflammatory and neurodegenerative processes in MS patients (Zostawa et al., 2017; Huhn et al., 2019). Furthermore, it has been shown that sodium accumulation leads to the release of calcium, which can exacerbate neurological disorders (Yang et al., 2015).

**Table 5:** Top 10 most enriched KEGG pathways.

**KEGG**	**Enriched shortest paths**	**Functions**	**Experimental method**	**References**
B CELL RECEPTOR SIGNALING PATHWAY	50	Survival and priming of B cells to receive T-cell help and B cells may potential contributors to progressive MS.	Flow cytometry	Chen et al., 2023; Touil et al., 2023
HEMATOPOIETIC CELL LINEAGE	36	Impact on immune function and bone marrow hematopoiesis might drive MS progression	Single-cell sequencing	Gao et al., 2018; Wu and Zhou, 2022
COMPLEMENT AND COAGULATION CASCADES	29	Complement and coagulation were major blood-borne proteolytic cascades and might play a role in the pathogenesis of MS.	an animal model of MS, Single-cell RNA sequencing and flow cytometry	Conway, 2018; Plantone et al., 2019
MAPK SIGNALING PATHWAY	16	Critical for cell survival and proliferation, cell adhesion and chemotaxis, and pro-inflammatory responses of immune cells.	xMAP Assays, flow cytometry and ELISA	Kotelnikova et al., 2019; Ten Bosch et al., 2021
NEUROTROPHIN SIGNALING PATHWAY	12	Associated with neuronal survival, development and function.		Reichardt, 2006; Bohmwald et al., 2022
NEUROACTIVE LIGAND RECEPTOR INTERACTION	12	1) Closely related to neurological function 2) Impact on memory capacity.		Reichardt, 2006
CELL ADHESION MOLECULES CAMS	9	1) In the immune system, CAMs regulated cell development, activation, differentiation, migration and many other cellular processes of crucial importance for the immune response. 2) Involved in the regulation of synaptic plasticity and the formation of neuronal networks. 3) CEACAM1 might prove to be a novel target for immunotherapy of multiple sclerosis.	Flow Cytometry	Lasky, 1991; Fujita et al., 2009; O'Callaghan et al., 2017; Guerra-Espinosa et al., 2024
ENDOCYTOSIS	7	1) Related to immunity, inflammation. 2) Phagocytosis was one of the prerequisites for myelin regeneration in MS patients.	Animal models fed CPZ	Sen et al., 2022
RENAL CELL CARCINOMA	6	Renal cell carcinoma and MS share the same risk factors age.		Bahadoram et al., 2022
PANCREATIC CANCER	6	MS was a risk factor for pancreatic cancer.		Liu Q. et al., 2023

The top ten BP terms were shown in Table 6 (Lasky, 1991; Rønn et al., 1998; Olivieri et al., 2003; Vély and Vivier, 2005; Suzuki and Takeichi, 2008; Shishido et al., 2012; Janssens et al., 2015; Yan et al., 2018; Nick, 2019; Bjornevik et al., 2023; Marzvanyan and Alhawaj, 2023; Soldan and Lieberman, 2023) and Figure 3 and Supplementary Figure S1 (without the starting point). For example, the BP term with the most enriched shortest paths was “VIRAL PROCESS” (GO: 0016032, enriched 53 shortest paths, Figure 3B and Supplementary Figure S1B). Some studies have shown that EBV is one of the primary causes of MS (Bjornevik et al., 2023), by affecting the cellular homeostasis (Sun et al., 2013). The BP term with minimum FDR was “REGULATION OF MEMBRANE REPOLARIZATION DURING VENTRICULAR CARDIAC MUSCLE CELL ACTION POTENTIAL” (GO:1905024, FDR = 0.0104, Figure 3D and Supplementary Figure S1D), which affected neurons, and thus MS, through action potentials (Mangold et al., 2017).

**Table 6:** Top 10 most enriched BP pathways.

**BP**	**Enriched shortest paths**	**Functions**	**Experimental method**	**Reference**
VIRAL PROCESS (GO:0016032)	53	EBV was an important factor in the cause of MS.	EB-virus immortalized B lymphocytes model	Olivieri et al., 2003; Bjornevik et al., 2023; Soldan and Lieberman, 2023
BIOLOGICAL PROCESS INVOLVED IN SYMBIOTIC INTERACTION (GO:0044403)	52	Symbiosis related to metabolism and immunity	Synchrotron micro-X-ray fluorescence	Nick, 2019
HOMOPHILIC CELL ADHESION VIA PLASMA MEMBRANE ADHESION MOLECULES (GO:0007156)	49	1) For synaptogenesis, not only stabilizes intercellular contacts at excitatory synapses but also assembles synaptic molecules at synaptic sites. 2) Involved in synaptic plasticity. 3) influences cell migration, neurite extension, and fasciculation.	Flow Cytometry	Rønn et al., 1998; Suzuki and Takeichi, 2008
CELL CELL ADHESION VIA PLASMA MEMBRANE ADHESION MOLECULES (GO:0098742)	49	Involved in the regulation of synaptic plasticity and the formation of neuronal networks.	Flow Cytometry	Lasky, 1991
IMMUNE RESPONSE REGULATING CELL SURFACE RECEPTOR SIGNALING PATHWAY (GO:0002768)	48	Associated with activation, perpetuation, or suppression of immune responses.		Vély and Vivier, 2005
IMMUNE RESPONSE REGULATING SIGNALING PATHWAY (GO:0002764)	48	Associated with activation, perpetuation, or suppression of immune responses.	Immunostaining and X-gal staining	Yan et al., 2018
REGULATION OF IMMUNE EFFECTOR PROCESS (GO:0002697)	47			
SENSORY ORGAN MORPHOGENESIS (GO:0090596)	46	They were responsible for helping maintain homeostasis in the body and for allowing the body to best react to internal and external events.		Marzvanyan and Alhawaj, 2023
HUMORAL IMMUNE RESPONSE (GO:0006959)	45	The humoral immune system played a role in the initiation and regulation of the inflammatory response.		Shishido et al., 2012
LEUKOCYTE MEDIATED IMMUNITY (GO:0002443)	42	IL-6 was associated with the pathogenesis of MS	Animal model of MS: IL-6R blockade	Janssens et al., 2015

The top ten KEGG pathways were shown in Supplementary Table S5 (containing the starting point) (Lasky, 1991; Reichardt, 2006; Allen et al., 2007; O'Callaghan et al., 2017; Conway, 2018; Gao et al., 2018; Kotelnikova et al., 2019; Plantone et al., 2019; Cui et al., 2020; Amoriello et al., 2021; Balkan and Bilge, 2021; Wu and Zhou, 2022; Chen et al., 2023; Leonard and Lin, 2023; Suptela and Marriott, 2023; Touil et al., 2023; Guerra-Espinosa et al., 2024). For example, one enriched pathway was “COMPLEMENT AND COAGULATION CASCADES,” where complement and coagulation were major blood-borne proteolytic cascades (Conway, 2018). Another crucial enriched pathway was “MAPK SIGNALING PATHWAY,” which was critical for cell survival, proliferation, adhesion and chemotaxis, as well as pro-inflammatory responses of immune cells (Kotelnikova et al., 2019). In addition, studies have shown that microglia with overactive MAPK interfere with local oligodendrocytes, leading to localized regional demyelination (a hallmark of MS) (Ten Bosch et al., 2021). The KEGG pathway with minimum FDR (Supplementary Table S6) was “NICOTINATE AND NICOTINAMIDE METABOLISM” (FDR = 0.001626), which produced the biologically active coenzymes, nicotinamide adenine dinucleotide (NAD), and its phosphate analog, the nicotinamide adenine dinucleotide phosphate (NADP) (Gasperi et al., 2019). Moreover, nicotinamide had been implicated in the development, survival and other function of neurons in the CNS, with both neuronal death and neuroprotection (Fricker et al., 2018). The top ten BP terms were shown in Supplementary Table S7 (Holman et al., 2011; Shishido et al., 2012; Zevini et al., 2017; Díaz et al., 2019; Silk and Margolin, 2019; Liu et al., 2022a,b; Huang et al., 2023) and the top ten BP terms with minimum FDR were shown in Supplementary Table S8 (containing the starting point). There were some BP terms connected to the microglia cell, which closely related to remyelination (Lloyd et al., 2019).

In summary, the enrichment analysis revealed various risk factors in MS, such as inflammation, neurodevelopment and cellular homeostasis.

### 2.5 Network markers identified potential risk markers

**Figure 4:**
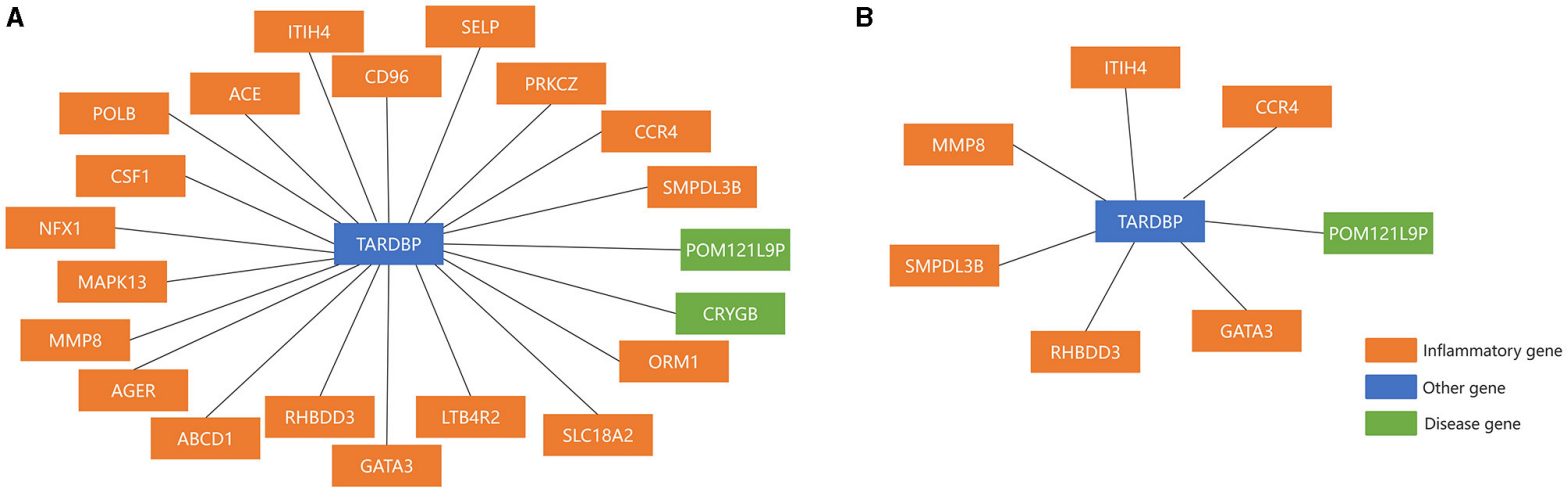
The top Network marker with the maximum betweeness. **(A)** Before sensitivity analysis; **(B)** after sensitivity analysis.

Network markers were identified by calculating the betweeness of the shortest path for each “inflammation-disease” pair, where the top 10 markers were shown in Table 7. For example, the top network marker (with the maximum betweeness and significant permutation result) was TARDBP (Transactive response DNA binding protein), where the relative network modules (including all the related shortest paths) were shown in Figure 4. It has been reported that TARDBP encoded the intranuclear protein TDP-43 (Transactive response DNA binding protein of 43 kDa) that played a role in the cellular stress response (Higashi et al., 2013). Stress granules were cytoplasmic foci that respond to cellular stress, and TDP-43 bound to ribosomes in stress granules, temporarily halting translation and promoting cytoprotective protein synthesis (Higashi et al., 2013; Baradaran-Heravi et al., 2020; Meneses et al., 2021). In addition, TARDBP was a risk factor for amyotrophic lateral sclerosis, frontotemporal dementia, and Alzheimer's disease, exacerbating cognitive impairment (Manohar et al., 2009; Meneses et al., 2021). In short, potential crucial risk markers were further identified by network analysis.

**Table 7:** Top 10 genes with the highest betweenesses.

**Before sensitive analysis**			**After sensitive analysis**		
**Gene Symbol**	**Betweenness**	***p*** **-value**	**Gene Symbol**	**Betweenness**	***p*** **-value**
TARDBP	21	0	TARDBP	6	0
VWA1	12	0	RNF207	3	0
GABRD	12	0	SCNN1D	2	0
RER1	11	0	PUSL1	2	0
RNF207	11	0	FAM43B	2	0
SAMD11	10	0	KLHL17	1	0
SCNN1D	9	0	VWA1	1	0
KCNAB2	6	0	GABRD	1	0
PUSL1	4	0	RER1	1	0
TP73	4	0	PHF13	1	0

### 2.6 Pan-cancer analysis further validated the mechanism of inflammaging in MS

**Figure 5:**
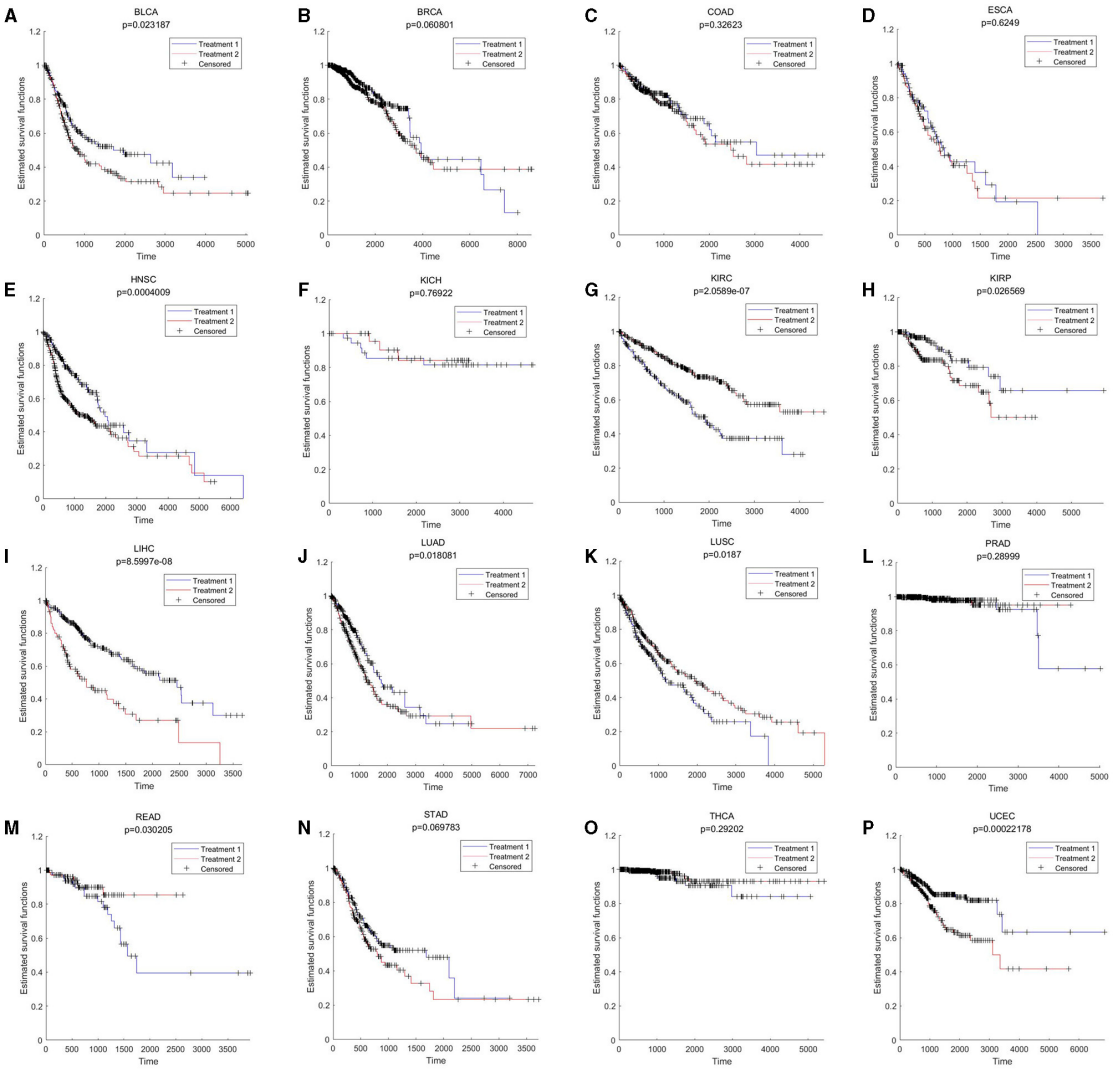
Survival results across different cancer types. BLCA, KICH, KIRC, PRAD, READ, STAD, THCA, and UCEC are based on aging markers; BRCA, COAD, ESCA, HNSC, KIRP, LIHC, LUAD, and LUSC are based on gene the whole differential co-expression pattern (by using Equations 9, 10 in Section 5.3). **(A)** BLCA; **(B)** BRCA; **(C)** COAD; **(D)** ESCA; **(E)** HNSC; **(F)** KICH; **(G)** KIRC; **(H)** KIRP; **(I)** LIHC; **(J)** LUAD; **(K)** LUSC; **(L)** PRAD; **(M)** READ; **(N)** STAD; **(O)** THCA; and **(P)** UCEC.

Pan-cancer analysis was used to further validate the relevant functions of “aging-inflammation-disease” triples. For example, the markers in triples were used to assess the survival indices across different cancer types. There were 9 out of 16 cancer types with significant results (including BLCA, HNSC, KIRC, KIRP, LIHC, LUAD, LUSC, READ and UCEC, shown in Figure 5). These results suggested that inflammaging markers could also be used as relative risk factors for cancer.

**Figure 6:**
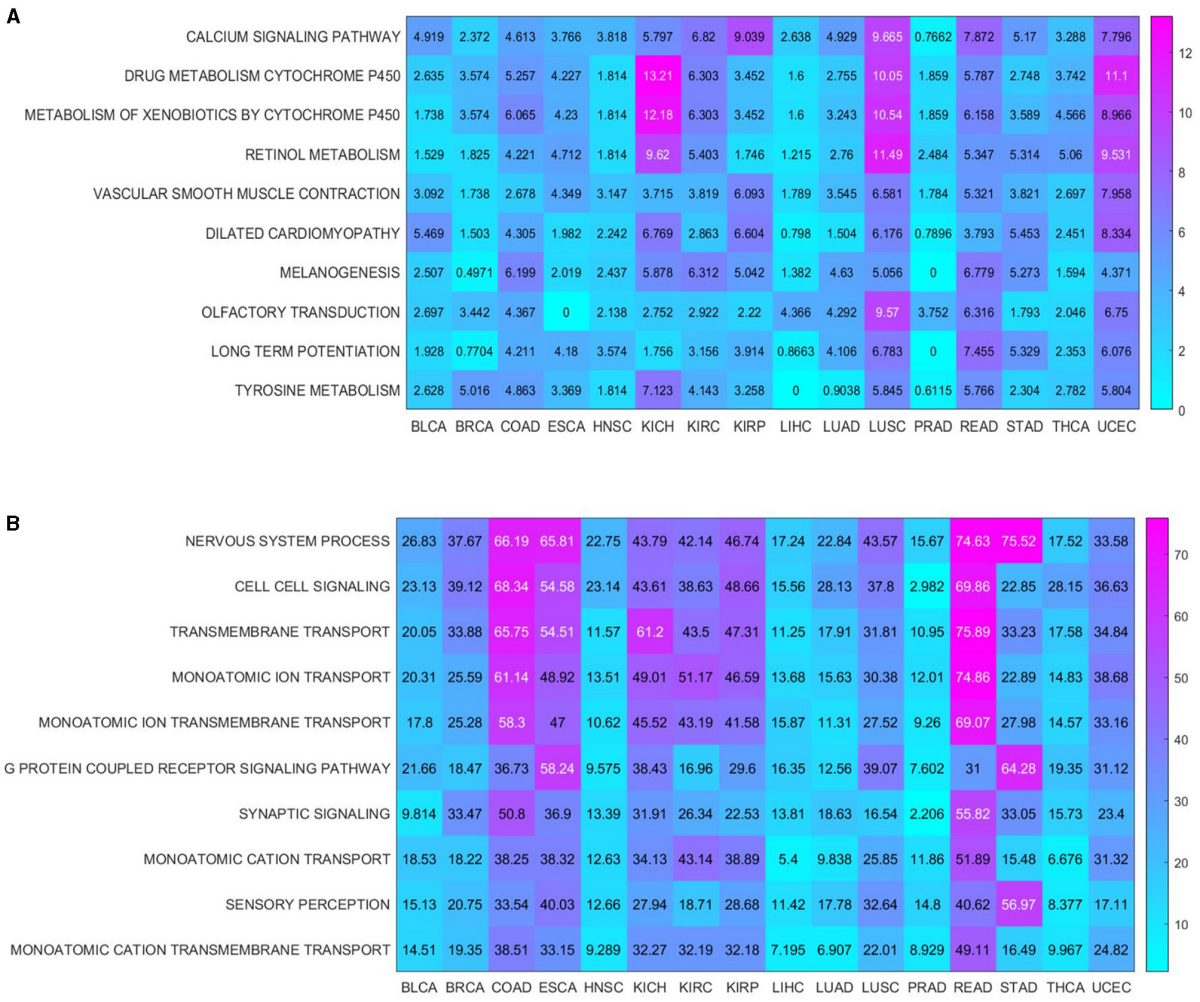
Enrichment analysis shared by cancers. **(A)** KEGG pathways enriched in 16 cancers; **(B)** BP terms enriched in 16 cancers.

In addition, based on enrichment analysis, both the commonality and specificity across 16 cancer types were further investigated. The top 10 common KEGG pathways were shown in Figure 6A, with the highest enrichment score of “CALCIUM SIGNALING PATHWAY.” A growing body of research suggested that calcium homeostasis contributed to well-known cancer-causing signals. Many studies had emphasized that calcium signaling contributed to the progression of several cancer types (e.g., glioma, prostate, and breast) through the activation of STAT3 (Wu et al., 2021). Meanwhile, calcium channels played an important role in the excitation and propagation of neuronal action potentials (Pourtavakoli and Ghafouri-Fard, 2022). The top 10 common BP terms were shown in Figure 6B, with the highest enrichment scoring of “NERVOUS SYSTEM PROCESS” (GO:0050877), indicating the key role of the nervous system in cancer (Hanahan and Monje, 2023). Supplementary Table S9 (Peterson et al., 2020; Feng et al., 2021; Glorieux and Buc Calderon, 2021; Hou et al., 2021; Naghshi et al., 2021) and Supplementary Table S10 (Martens and Mithöfer, 2005; Turner and Grose, 2010; Menezes et al., 2018; Arneson and Doles, 2019; Przygodzka et al., 2019; Keough and Monje, 2022; Ohkuni et al., 2022; Libretti and Aeddula, 2023; Lustberg et al., 2023) summarized the specific enrichment results in each cancer, indicating that a series of risk factors (such as neurodevelopment and cellular homeostasis) were also crucial to cancer.

In summary, our findings highlighted a series of key functions associated with inflammaging that could also be used to investigate potential mechanisms in cancer.

## 3 Discussion

The inflammatory response plays a crucial role in MS. However, the important relationships among aging, inflammation and MS remain to be further explored in depth. In this paper, a series of computational methods were used to explore these relationships and relative mechanisms in MS. First, both aging and disease predictors were modeled to identify relative aging and disease markers, respectively. Then, an integrated inflammaging model was developed to find important “aging-inflammation-disease” triples. Further, the potential mechanisms between inflammation and MS were investigated using network analysis, sensitivity analysis, enrichment analysis and pan-cancer analysis. In short, various risk factors in MS were integrated at system level.

Our findings emphasized that protein homeostasis was vital to the MS development. For example, the disease marker FNDC4, could lead to AMP-activated protein kinase (AMPK) phosphorylation (Lee et al., 2018). Moreover, in the MCMC (Table 3), the most sensitive marker of aging was TMPRSS13, playing a key role in proteolytic activity and phosphorylation (Martin et al., 2021). In the enrichment analysis, the KEGG pathway was “COMPLEMENT AND COAGULATION CASCADES,” which was associated with protein catabolism (Conway, 2018). In summary, our results also confirmed that the protein homeostasis played an important role in MS by interacting with the immune system, even accelerating the progression of MS (Negrotto and Correale, 2017).

The cellular homeostasis was also highlighted in this work. For example, the top network marker was TARDBP (Figure 4), which encoded the intranuclear protein TDP-43 that played a role in the cellular stress response (Higashi et al., 2013). Moreover, in MCMC (Table 3), the inflammatory marker CUEDC2 was involved in the cell cycle regulation (Xiao et al., 2019). According to the enrichment analysis, one of the most enriched KEGG pathways was “MAPK SIGNALING PATHWAY.” The MAPK pathway is associated with cell proliferation, differentiation, migration, senescence, and apoptosis (Sun et al., 2015). Furthermore, lytic cell death pathways (such as pyroptosis, necroptosis, ferroptosis, and PANoptosis) are closely related to neuroinflammation and even exacerbate MS (Lee et al., 2023). This demonstrated the key role of cellular homeostasis in MS.

Neurodevelopment played an important role in the development of MS. For example, the top aging marker TSPAN6 was a regulator of synaptic transmission and plasticity mechanisms (Salas et al., 2017). The inflammaging model had identified a series of inflammatory markers associated with neuronal formation in MS (Table 2): DO1 had a role in regulating neuronal excitability (Correale, 2021); SLC18A2 was neuroprotective and PNMA1 promoted neuronal apoptosis (Chen and D'Mello, 2010; Black et al., 2021). In the enrichment analysis, the KEGG pathways “NEUROTROPHIN SIGNALING PATHWAY” and “NEUROACTIVE LIGAND RECEPTOR INTERACTION” also highlighted the neurodevelopment (Reichardt, 2006; Bohmwald et al., 2022). MS is a wellknown neuroinflammatory disease, where neuronal damage is vital to the progression of MS lesions (Schirmer et al., 2019). In sum, our results highlighted the neurodevelopment in MS.

The energy metabolism was also involved in the development of MS. For example, in the integrated inflammaging model (Table 2), the aging marker BMP8A, enabled anti-adiposity by promoting fatty acid oxidation and inhibiting adipocyte differentiation (Zhong et al., 2023); the inflammatory marker IGFBP4 was an important regulator of adipose tissue development (Maridas et al., 2017). In addition, in MCMC (Table 4), the inflammation marker SHPK catalyzed the phosphorylation of sedoheptulose in the non-oxidative arm of the pentose phosphate pathway (Franceschi et al., 2022). Furthermore, in the enrichment analysis (Supplementary Table S6), the KEGG pathway with minimum FDR was “NICOTINATE AND NICOTINAMIDE METABOLISM” (FDR = 0.001626), which produced the biologically active coenzymes NAD (its phosphate analog was the NADP) (Gasperi et al., 2019). There was also a series of pathogenesis in MS along with the energy failure of the CNS (Park and Choi, 2020). These results suggested that energy metabolism was closely related to the MS progression.

It has been well known that MS is a chronic inflammatory disease, which is closely related to the aging process. Herein various risk factors for MS were explored based on aging-related inflammation (inflammaging). In this work, a series of computational methods were used to investigate potential molecular mechanisms in MS. An inflammaging model was constructed to obtain the “aging-inflammation-disease” triples, and then crucial inflammaging characteristics in MS were identified. In addition, these results could also indicate further the relative experimental validations. In short, the complex mechanisms in MS could be further studied by exploring key inflammaging indices, where various risk factors were integrated at system level.

Stridently, the identified inflammaging characteristics in MS (i.e., the inflammaging markers, enriched KEGG pathways or BP terms, shown in Tables 2–6) have been validated by a series of relative experiment results. For example, inflammaging could alter the transport capacity of B cells, making them more sensitive to cytokines and pro-inflammatory molecules, which were overproduced in the elderly (Bulati et al., 2014). Recently, using the flow cytometry, it has been demonstrated that the combination of pro-inflammatory interleukin-21 (IL-21) and B-cell receptor (BCR) stimulation enabled B cells to produce/secrete the active form of the cytotoxic serine protease granzyme B (GrB), which might exacerbate the MS progression (Niland et al., 2010; Bulati et al., 2014). Further, the coagulation pathway was also identified in this work, and even confirmed by other experimental results of MS (by using animal models, single-cell RNA sequencing, or flow cytometry). It has been reported that the coagulation cascade increased neuroinflammation during the aging process, thus interacting with a series of physiological factors such as neuronal deficits, oxidation, or dysfunction of the endoplasmic reticulum and mitochondria, which in turn contributed to the onset of MS (Conway, 2018; Plantone et al., 2019). In addition, Enzyme-linked immunosorbent assay (ELISA) indicated that “Bovine serum albumin (BSA)-advanced glycation end (AGE)” enhanced IL-6 expression through MAPK-ERK action (MAPK-ERK and MyD88 transduced NF-κB signaling pathways), and studies (both *in vitro* and *in vivo*) have demonstrated that IL-6 played a crucial role in regulating the immune response in MS (Janssens et al., 2015; Shen et al., 2019). The EBV infection have also been reported to increase the risk of developing MS approximately 32-fold (Bjornevik et al., 2022). EBV infected of B cells and T cells, leading to infected B cells infiltrated of the CNS and T cell exhaustion, where CD8 T cell deficiency contributed to the decreased CD8 T cell response to EBV-infected B cells and with functional declined in aged MS patients (Pender et al., 2012; Soldan and Lieberman, 2023). Note worthily, the perpetuation of “forbidden” autoreactive B-cell clone by EBV immortalization have been suggested as a potential mechanism for triggering MS (Pender, 2011). For example, in the context of inflammaging and immunosenescence, EB-virus immortalized B lymphocytes model have been shown to produce higher levels of IL-6, which was associated with the pathogenesis of MS (Olivieri et al., 2003; Janssens et al., 2015). In short, a series of key risk factors in MS were identified based on inflammaging, and even could be confirmed by relative experiments.

Studies had shown that the risk of cancer was increased in people with MS (Ragonese et al., 2017; Bosco-Lévy et al., 2022). It was well known that MS and cancer shared a series of common risk factors such as immunity, inflammation, cellular homeostasis, neurodevelopment, and protein homeostasis (Davidson et al., 2017; Przygodzka et al., 2019; Singh et al., 2019; Miller and Thorburn, 2021). Our findings also demonstrated that inflammation, neurodevelopment and cellular homeostasis were common risk factors in cancer [Figure 6B, i.e., “NERVOUS SYSTEM PROCESS” (GO:0050877), “SYNAPTIC SIGNALING” (GO:0099536) and “CELL CELL SIGNALING” (GO:0007267)]. Additionally, the key roles of inflammaging markers in different cancer types were further confirmed by survival analysis. Inflammation was increasingly recognized as an important factor impairing normal functions in CNS, which in turn affected both cancer and MS (Deverman and Patterson, 2009; Jiang et al., 2018). In addition, chronic inflammation disrupted the cellular homeostasis, which played an important role in the development of both MS and cancer (Kotas and Medzhitov, 2015). In short, various risk factors associated with inflammaging had also been demonstrated in cancer.

As with other research articles on MS (Denissen et al., 2021; Aslam et al., 2022) and other neurodegenerative diseases [e.g., Alzheimer's disease (Chang et al., 2021; Li J. et al., 2022) and Parkinson's disease (Boutet et al., 2021; Oliveira et al., 2023)], machine learning was utilized to build high accuracy models or predictive biomarkers, which were then subjected to enrichment analysis network analysis and so on. In addition, our study identified an integrative model based on machine learning to further explore the underlying mechanisms of MS in the context of inflammaging. As a result, a series of relative key risk factors were summarized at system level, and even validated across different cancer types. These results indicated that our results were with enough reliability and accuracy.

**Figure 7:**
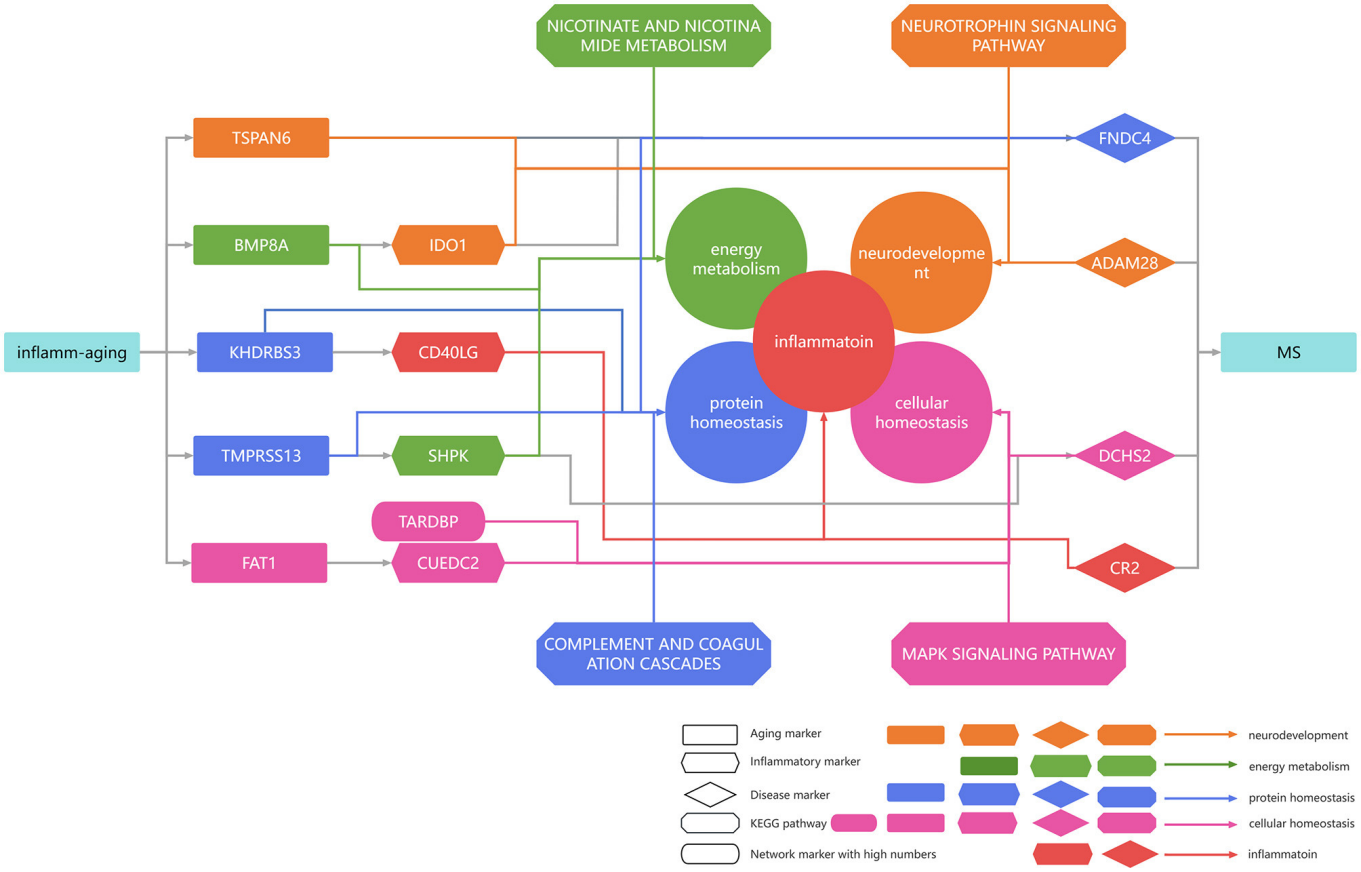
Summarized mechanisms in multiple sclerosis in the context of inflammaging. Rectangular genes represent aging markers, diamond genes represent disease markers, rhombic genes represent disease markers, and oval genes represent high median network markers. Orange arrows indicate that the gene was associated with neurodevelopment, green arrows indicate that the gene was associated with energy metabolism, blue arrows indicate that the gene was associated with protein homeostasis, pink arrows indicate that the gene was associated with cellular homeostasis, and red arrows indicate that the gene was associated with inflammation.

According to the inflammaging theory, the chronic inflammation was accumulated during the aging process, along with a series of dysregulated pathways (Fang et al., 2018). In addition, the immunosenescence was also accompanied with a series of molecular dysfunctions in both innate and adaptive immune systems, and even interacted with aging (Rodrigues et al., 2021; Liu et al., 2023). Both inflammation and aging were wellknown to affect microglia and astrocytes, which in turn impaired normal neurons (Neumann et al., 2019; Kwon and Koh, 2020; Diaz-Castro et al., 2021). Inflammation also affects the protein metabolism and cellular homeostasis (Antonangeli et al., 2021; Cibrian et al., 2022). In addition, these risk factors interplayed with each other to promote the development of MS. For example, dysregulations in cellular homeostasis can interact with the protein homeostasis, energy metabolism, etc., which in turn aggravated the MS progression (Huang et al., 2022). With the help of the integrated inflammaging model, our study highlighted a series of risk factors closely related to inflammaging in MS, such as protein homeostasis, cellular homeostasis, neurodevelopment and energy metabolism. These results also further confirmed both the theories of inflammaging and immunosenescence (Figure 7). In short, we integrated the potential mechanisms of MS in the context of inflammaging (Figure 7).

Despite the exploration of the underlying mechanisms in MS based on the inflammaging, there were still shortcomings as follows: (1) This paper only used 445 samples of microarray profiles, where the single-cell profiles should investigated in further analysis; (2) The biological experiments were still vital to performed to further validate relative conclusions in human cell line, if with proper permissions; (3) The potential mechanisms of MS were identified only based on inflammaging, without considering other key risk factors (e.g., oxidative stress or neuroendocrine). After all, a series of investigations were still needed to further explore underlying mechanisms in MS (or other neuroinflammatory diseases), where our work presented a novel thought to study relative molecular mechanisms.

## 4 Conclusion

In this study, machine learning was used to construct models for predicting aging and disease (MS) and to identify relative biomarkers. The important relationship between inflammaging and MS was further explored by building the integrated inflammaging model. Relative inflammaging characteristics in MS patients were investigated holistically through sensitivity, enrichment, network and pan-cancer analyses. In summary, our study integrated protein homeostasis, cellular homeostasis, neurodevelopment and energy metabolism as risk factors in MS based on inflammaging indices, also presenting a novel thought to other aging-related diseases.

## 5 Materials and methods

### 5.1 Data and preprocessing

The gene expression data were downloaded from the Gene Expression Omnibus (GEO) database (https://www.ncbi.nlm.nih.gov/geo/), including GSE190847, GSE131281, GSE126802, GSE108000, GSE78244, GSE37750, GSE62584, GSE41890, and GSE14895. These datasets were from eight different microarray platforms: GPL23126, GPL10558, GPL13497, GPL17077, GPL570, GPL571, GPL6244, and GPL96.

The gene expression profiles were processed as follows:

(1) Only the samples with both the age and phenotype indices (MS or control) were retained; otherwise, they were excluded.
(2) The gene expression matrix for each dataset was integrated by summarizing the probe number within the gene symbol.
(3) The total data matrix was integrated, and the missing gene expression values were filled with values of 0.
(4) Data processing was performed on the summary matrix to remove genes with ≥30% missing values.
(5) The gene expression matrix was logarithmically transformed if it contained outliers.
(6) Based on the mean and standard deviation of gene expression for the control individuals, z-score normalization was performed for both the MS and control samples.
(7) The singular value decomposition (SVD) method was performed to eliminate the inter sample variation based on the top three principal components of the control samples.
(8) The z-score was then utilized to normalize all samples based on the mean and the standard deviation of the control samples.
(9) The gene expression profiles were further transformed using the hyperbolic tangent (Tanh) method, so that it takes values between −1 and 1.
(10) The training set and the test set were randomly divided at a ratio of approximately 2:1.

As a result, a total of 445 samples were obtained (Supplementary Table S1), including 66 samples of healthy aged people (aged ≥ 50 years, 45 training datasets +21 test datasets), 118 samples of healthy young people (aged <50, 80 + 38), 94 samples of MS aged people (aged ≥ 50, 65 + 29) and 167 samples of MS young people (aged <50, 115 + 52), containing 16,275 gene symbols (Supplementary Table S2). Further, comparison results based on the inter-sample normalization step (i.e., z-score, SVD, and another z-score) have been show in Supplementary Figure S2, including boxplots and scatter plots. These results indicated that the normalization could treat relative profiles from different platforms with enough efficiencies, indicating that the profiles were clustered with each dataset before the normalization, comparable after the normalization, and even distinguishable between different phenotypes (i.e., MS or control) if combining with machine learning methods.

We also obtained paired gene expression (RNAseq) profiles (“Batch effects normalized mRNA data”) and clinical data from the TCGA database through the xena platform (https://xenabrowser.net/hub/). Cancer types with ≥10 adjacent normal samples were retained. There were 16 cancer types included in this study: BLCA (408 cancer samples and 19 adjacent normal samples), BRCA (1,102 + 113), COAD (451 + 41), ESCA (185 + 11), HNSC (522 + 44), KICH (66 + 25), KIRC (534 + 72), KIRP (291 + 32), LIHC (373 + 50), LUAD (517 + 59), LUSC (504 + 51), PRAD (498 + 52), READ (160 + 10), STAD (414 + 35), THCA (513 + 59), and UCEC (533 + 22). Genes with ≥30% missing values were deleted.

### 5.2 Modeling the aging model and disease model

The ReliefF algorithm was used to select key features, and then the first 100 models were studied to train the predictors. The optimal model was selected by 10-fold cross-validation. To verify the accuracy of the aging predictor, the selected model was verified in the test dataset.

(1) In the aging model, the normal aged group (age ≥ 50) was labeled 1, and the young healthy group (age <50) was labeled 0; in the disease model, the MS group was labeled 1, and the control group was labeled 0.
(2) The ReliefF algorithm was used to sort 16,275 genes for the aging and disease models;
(3) The predictor of the model was used to select the key markers with the help of the k-nearest neighbors (kNN, k = 9, correlation) algorithm. The model with the highest accuracy was also selected with the help of 10-fold cross-validation, where the identified features were considered aging markers and disease markers.

As a result, the optimal k-nearest neighbor (kNN, k = 9, correlation) algorithm was used, and a total of 70 aging markers and 19 disease markers were identified. In addition, these markers could be summarized as the aging score and disease score for further analyses (i.e., comparison in Section 5.1 or sensitive analysis in Section 5.4).


(1) $$\begin{array}{c} \text{aging\_score}=\overset{5}{\underset{\text{k}=1}{\sum }}\text{distance\_of\_nearst\_neighbor\_in\_normal\_young}\\ -\overset{5}{\underset{\text{k}=1}{\sum }}\text{distance\_of\_nearst\_neighbor\_in\_normal\_old} \end{array}$$

(2) $$\begin{array}{c} \text{disease\_score}=\overset{5}{\underset{\text{k}=1}{\sum }}\text{distance\_of\_nearst\_neighbor\_in\_control}\\ -\overset{5}{\underset{\text{k}=1}{\sum }}\text{distance\_of\_nearst\_neighbor\_in\_MS} \end{array}$$

As a result, the ROC curve could be designed based on the aging and disease score, respectively.

### 5.3 Identifying essential relationships in MS by integrating inflammaging models

An integrated inflammaging model was built to identify the essential relationships among aging, inflammation and MS. The computational pipeline used was MR, although it was not as rigorous as MR (Burgess et al., 2020, 2023).

In this model, the aging-related inflammatory markers were considered inflammaging markers, where candidate aging/disease markers were identified in Section 5.2 to be further select relate to these inflammaging markers. Ultimately, the essential relationships among aging, inflammation, and disease (MS) markers were identified as key “aging-inflammation-disease” triples in MS.

Here, the aging markers were used as the auxiliary variables (similar to the instrumental variables in MR), and the inflammatory markers were used as the candidate risk factors. Then, inflammatory (“inflammaging”) markers were identified as the risk factors, and disease markers were used as the outcome variables. That is, the integrated inflammaging model aimed to explore the essential relationships among aging, inflammation and disease markers in MS. The objectives of the “aging-inflammation-disease” triples were as follows:

(1) There was a correlation between the aging marker and the inflammatory marker.
(2) There was a correlation between the inflammatory marker and the disease (MS) marker.
(3) There was a correlation between the aging marker and the disease (MS) marker.
(4) There was a strong correlation between the aging marker and the disease marker, if through the inflammatory marker.

The methodological steps of the model were as follows:

(1) The inflammatory markers used as candidate risk factors were obtained through the Gene Set Enrichment Analysis (GSEA) platform based on the biological process (BP) of gene ontology (GO) (http://www.gsea-msigdb.org/gsea/downloads.jsp, version 2023.1, with “INFLAMMATORY” as keywords). As a result, 745 candidate inflammatory markers were selected.
(2) The correlation (differential co-expression) pattern was used to select aging markers that strongly correlated with candidate inflammatory markers with the help of the Kruskal–Wallis test. Here, the differential co-expression was calculated as follows:

(3) $$\begin{array}{c} \begin{array}{c} \text{p}=\text{Kruskal}-\text{Wallis test}\\ \left(\text{aging\_marker}.*\text{inflammation\_marker},\text{phenotype}\right) \end{array} \end{array}$$

where the phenotype could be defined as 1 (MS) or 0 (control).

Furthermore, both a *p*-value < 0.05 and a Benjamini–Hochberg false discovery rate (FDR) < 0.1 were used to select strongly correlated aging markers.

(3) The correlation (differential co-expression) was used to select inflammatory markers that strongly correlated with disease markers with the help of the Kruskal–Wallis test. Here, the differential co-expression was calculated as follows:

(4) $$\begin{array}{c} \begin{array}{c} \text{p}=\text{Kruskal}-\text{Wallis test}\\ \left(\text{inflammation\_marker}.*\text{disease\_marker},\text{phenotype}\right) \end{array} \end{array}$$

where the phenotype could be defined as 1 (MS) or 0 (control).

Furthermore, both a *p*-value < 0.05 and a Benjamini–Hochberg false discovery rate (FDR) < 0.1 were used to select strongly correlated inflammatory markers.

(4) The correlation (differential co-expression) was used to select disease markers that strongly correlated with aging markers with the help of the Kruskal–Wallis test. Here, the differential co-expression was calculated as follows:

(5) $$\begin{array}{c} \text{p}=\text{Kruskal}-\text{Wallis test}\\ \left(\text{disease\_marker}.*\text{aging\_marker},\text{phenotype}\right) \end{array}$$

where the phenotype could be defined as 1 (MS) or 0 (control).

Furthermore, both a *p*-value < 0.05 and a Benjamini–Hochberg false discovery rate (FDR) < 0.1 were used to select strongly correlated disease markers.

(5) To filter out the effect of horizontal pleiotropy, the aging–disease relationship was further examined by comparing the correlation between each aging marker and disease marker through the inflammatory marker or otherwise. Here, steps ①-③ were used to calculate the correlations between auxiliary variables and outcome variables without the background of the risk factor, and step ④ was used to calculate the correlations between auxiliary variables and outcome variables with the context of the risk factor.

① The residual of each disease marker (“residual A”) was calculated based on the inflammatory marker:

(6) $$\begin{array}{c} {\text{residual\_A}=\text{disease\_marker}-\text{b}}_{1}*\text{inflammation\_marker} \end{array}$$

where *b1* was the regression coefficient.

② The residual of each aging marker (“residual B”) was calculated based on the inflammatory marker:

(7) $$\begin{array}{c} {\text{residual\_B}=\text{aging\_marker}-\text{b}}_{2}*\text{inflammation\_marker} \end{array}$$

where *b2* was the regression coefficient.

③ The abovementioned two residuals were further compared, and the residual of the disease marker was calculated (as “residual C”):

(8) $$\begin{array}{c} {\text{residual\_C}=\text{residual\_A}-\text{b}}_{3}*\text{residual\_B} \end{array}$$

where b3 was the regression coefficient.

④ The residual disease marker (“residual D”) was calculated based on the aging marker.

⑤ The difference (between “residual C” and “residual D”) between the MS and control subgroups was tested using the Kruskal–Wallis test (*P* < 0.05 and FDR < 0.1).

Finally, the essential relationships among aging markers, inflammatory markers and disease markers were determined. Thus, 5,599 “aging-inflammation-disease” triplets were identified, including 65 aging markers, 107 inflammatory markers (as the 107 inflammaging markers) and 19 disease markers. Thus, these 107 inflammatory markers were used as inflammaging markers (risk factors), and 19 disease markers were also used to discriminate the MS phenotype.

In addition, the whole differential co-expression pattern among aging, inflammation and disease markers could be calculated based on these triples.


(9) $$\begin{array}{l} {\text{inflammation}}_{\text{i}}\\ =\sum \text{corr}({\text{inflammation}}_{\text{i}}^{\text{j}}.*{\text{aging}}^{\text{j}},\text{phenotype}) \end{array}$$

where i and j was the i-th inflammation marker and the j-th aging marker, *corr* was the Pearson's correlation coefficient, and the phenotype could be defined as 1 (MS) or 0 (control). The differential co-expression of a inflammation marker was summarized based the related aging markers in the triples.


(10) $$\begin{array}{l} {\text{disease}}_{\text{k}}\\ =\sum \text{corr}({\text{disease}}_{\text{k}}^{\text{i}}.*{\text{inflammation}}^{\text{i}},\text{phenotype}) \end{array}$$

where *k* and *i* was the k-th disease marker and the i-th inflammation marker, *corr* was the Pearson's correlation coefficient, and the phenotype could be defined as 1 (MS) or 0 (control). The differential co-expression of a disease marker was summarized based the related inflammation markers in the triples.

### 5.4 Sensitivity analysis using the MCMC method

To further explore crucial relationships among aging, inflammation and MS, sensitivity analysis was performed based on the Markov Chain Monte Carlo (MCMC) method, where “aging-inflammation-disease” triples were further evaluated as a candidate relationship. The MCMC method was used to sample certain posterior distributions in a high-dimensional space based on a given probabilistic background. The key step of MCMC was to construct a Markov chain whose equilibrium distribution was equal to the target probability distribution. The steps were as follows:

(1) Constructing the transfer cores of the ergodic Markov chain. The prior distribution of each parameter was normally distributed based on all identified markers in each group (i.e., MS or control), respectively.
(2) Simulate the chains until equilibrium was reached. The Metropolis–Hastings sampling method was used to determine whether the new sample (θ ^*^) was acceptable based on the α value.

(11) $$\begin{array}{c} \alpha =\frac{\text{p}{\left(\theta^{*}|\text{X}\right)}^{*}\text{q }\left(\theta^{\text{n}}\to \theta^{*}\right)}{\text{p}{\left(\theta^{\text{n}}|\text{X}\right)}^{*}\text{q }\left(\theta^{*}\to \theta^{\text{n}}\right)} \end{array}$$

where *P (*θ^*n*^ *| X)* and *P (*θ^*^| X) were the posterior probability of the nth accepted sample, the new sample q (θ^n^ → θ^*^) was the transition probability from the nth accepted sample to the new sample, and *q (*θ^*^ → θ^*n*^*)* was the transition probability from the new sample to the n-th accepted sample.

In this work, the disease score was used to evaluate the simulated samples, with 1,000 random samples used as candidate samples for each group (i.e., MS or control). The disease score was calculated by comparing the distance between normal and MS training samples based on the 19 disease markers identified by the integrated inflammaging model, by using the Equation (2).

(3) Performing the global sensitivity analysis

The correlation index was used to evaluate each of the “aging-inflammation-disease” triples in the accepted samples (both MS and control):


(12) $$\begin{array}{c} \text{correlation\_index}=\frac{\text{disease\_marker}-\text{aging\_marker}}{\text{inflammation\_marker}-\text{aging\_marker}} \end{array}$$

Therefore, the correlation indices were calculated for each “aging-inflammation-disease” triple for all accepted samples. Then, the Kruskal–Wallis test was used to evaluate each correlation index in each “aging-inflammation-disease” triple, where *p*-value < 0.05 and FDR < 0.1 were set as the threshold. Finally, a total of 35 “aging-inflammation-disease” triples were identified as sensitive relationships, including 16 aging markers, 28 inflammatory markers, and 9 disease markers.

### 5.5 Constructing the differential co-expression network

To further reveal potential mechanisms between “inflammaging” and MS, a differential co-expression network was constructed via the following steps:

(1) The Pearson correlation coefficient for each pair of genes was calculated based on the MS and control groups.
(2) The Benjamini–Hochberg FDR method was used to adjust the *p*-values of the correlation coefficients.
(3) The relationship between each gene pair was retained if the coefficient value in MS had the opposite sign (i.e., + or –) to that in the control, as well as if *p* < 0.05 and FDR < 0.1.
(4) The shortest path between each pair of inflammaging and disease markers was selected based on the differential co-expression network using the Dijkstra algorithm.

### 5.6 Enrichment analysis

The gene functions were further explored by enrichment analysis of the shortest pathway. Gene Ontology (GO) terms and KEGG pathways for the GSEA platform were obtained from gene set enrichment analysis (http://software.broadinstitute.org/gsea/downloads.jsp, version 2023.1). The hypergeometric distribution was used to test the degree of enrichment of the GO BP and KEGG pathways. Hypergeometric test formula:


(13) $$\begin{array}{c} \text{P}\left(\text{X}\geq \text{x}\right)=1-\overset{\text{x}-1}{\underset{\text{k}=0}{\sum }}\frac{{\text{C}}_{\text{M}}^{\text{k}}\times {\text{C}}_{\text{N}-\text{M}}^{\text{n}-\text{k}}}{{\text{C}}_{\text{N}}^{\text{n}}} \end{array}$$

where *N* was the total number of genes in the gene set, *M* was the number of known genes (such as the KEGG pathway or BP terms), which was the number of genes identified in each shortest pathway, and *k* was the number of common genes between known genes and candidate genes identified in each “inflammation-disease” shortest pathway. The *p-*value of each path was controlled using the Benjamin–Hochberg method. Finally, pathways with *p* < 0.05 and FDR < 0.1 were retained.

### 5.7 Identifying network markers

The subnetwork with the shortest pathways among the selected “inflammation-disease” pairs was constructed, and genes in the subnetwork were sorted by their betweennesses in descending order. To test whether the top betweenness genes were hubs in the background network, we ran a permutation to count the occurrence time of the top genes in the shortest paths between randomly selected genes (containing the same numbers of “inflammation-disease” triples, based on the identified “aging-inflammation-disease” triples) when they had greater betweennesses than those in our study. We repeated this process 1,000 times, and the *p*-value was calculated as the proportion of occurrence times of the top betweenness genes in 1,000 permutations.

### 5.8 Pan-cancer analysis

The survival analysis was performed based on the inflammaging markers (identified by the integrated inflammaging model in Section 5.3) for each cancer using the Kaplan–Meier method. The first principal component of the triples set of key markers for each cancer was taken, and then they were categorized into two groups based on the mean values. Then, the Kaplan–Meier method was used to evaluate the survival difference between these two groups, and the significance was estimated by the log-rank test. A *p*-value < 0.05 was considered statistically significant.

Genes were considered differentially expressed if they satisfied the following criteria:

(1) Fold change > 2;
(2) *p*-value < 0.05 according to the Kruskal–Wallis test;
(3) Benjamin-Hochberg false discovery rate (FDR) < 0.1.

Then, the differential expression networks were constructed for each cancer, where the details were also the same as Section 5.5. As a result, each shortest pathway was selected from each pair of inflammaging markers and differentially expressed genes (as disease markers in cancer) using the Dijkstra algorithm. Furthermore, the enrichment analysis was performed in each cancer (*p* < 0.05 and FDR < 0.1).

## Data availability statement

The original contributions presented in the study are included in the article/Supplementary material, further inquiries can be directed to the corresponding authors.

## Author contributions

MX: Conceptualization, Data curation, Formal analysis, Investigation, Methodology, Software, Validation, Visualization, Writing – original draft. HW: Conceptualization, Investigation, Software, Visualization, Writing – original draft. SR: Data curation, Formal analysis, Methodology, Validation, Writing – original draft. BW: Investigation, Validation, Visualization, Writing – review & editing. WY: Investigation, Validation, Visualization, Writing – review & editing. LL: Investigation, Validation, Visualization, Writing – original draft. XS: Conceptualization, Funding acquisition, Investigation, Project administration, Supervision, Writing – review & editing. WL: Conceptualization, Funding acquisition, Investigation, Project administration, Supervision, Writing – review & editing. YW: Conceptualization, Funding acquisition, Investigation, Methodology, Supervision, Validation, Writing – review & editing.

## Supplementary material

Supplementary Figure S1 Enrichment analysis of the shortest paths of KEGG and BP, before combining overlap shortest paths. **(A)** KEGG with the most shortest enriched paths; **(B)** BP with the most shortest enriched paths; **(C)** KEGG with the minimum FDR; **(D)** BP with the minimum FDR. The orange nodes represent the inflammaging markers, the blue nodes represent the genes connecting inflammaging markers and disease markers, the green nodes represent the disease markers, and the genes in the red square frames coincide with those genes in the enriched functions.

Supplementary Figure S2 Comparison results based on the inter-sample normalization step. **(A, B)** The boxplot of the control samples in each dataset, based on the first principal component; **(C, D)** the scatter plot in each dataset, based on the first two principal component; **(E, F)** the scatter plot in each dataset, based on the aging score and diease score; **(A, C, E)** before the normalization; **(B, D, F)** after the normalization.

Supplementary Table S1 Detailed datasets used in this work.

Supplementary Table S2 Gene symbols used in this work.

Supplementary Table S3 ReliefF ranking of aging markers.

Supplementary Table S4 ReliefF ranking of disease markers.

Supplementary Table S5 Top 10 most enriched KEGG pathways containing inflammatory markers.

Supplementary Table S6 The top 10 KEGG pathways with minimum FDR containing inflammatory markers.

Supplementary Table S7 Top 10 most enriched BP terms containing inflammatory markers.

Supplementary Table S8 The top ten BP terms with minimum FDR containing inflammatory markers.

Supplementary Table S9 KEGG pathways in each cancer with minimum FDR.

Supplementary Table S10 BP terms in each cancer with minimum FDR.
