# Supplementary material for: Identification of crucial inflammaging related risk factors in multiple sclerosis
Source: Front Mol Neurosci. 2024 May 21;17:1398665. doi: 10.3389/fnmol.2024.1398665 (PMC11148336; doi:10.3389/fnmol.2024.1398665)
Supplement: Supplementary Figure S1 — Enrichment analysis of the shortest paths of KEGG and BP, before combining overlap shortest paths. (A) KEGG with the most shortest enriched paths; (B) BP with the most shortest enriched paths; (C) KEGG with the minimum FDR; (D) BP with the minimum FDR. The orange nodes represent the inflammaging markers, the blue nodes represent the genes connecting inflammaging markers and disease markers, the green nodes represent the disease markers, and the genes in the red square frames coincide with those genes in the enriched functions. [file Data_Sheet_1.ZIP › Supplemental files/Table S5-S10.docx]

**Table S5** Top 10 most enriched KEGG pathways containing inflammatory markers

| KEGG | Enriched shortest paths | Functions | Reference |
| --- | --- | --- | --- |
| CYTOKINE CYTOKINE RECEPTOR INTERACTION | 62 | 1. Cytokines had a key role in immune system regulation and played a role in the course and pathogenesis of MS 2. Cytokines were associated with cell development, differentiation, growth and survival. | 104,105 |
| B CELL RECEPTOR SIGNALING PATHWAY | 54 | survival and priming of light zone B cells to receive T-cell help. | 69 |
| HEMATOPOIETIC CELL LINEAGE | 49 | Impact on immune function. | 71 |
| CHEMOKINE SIGNALING PATHWAY | 42 | 1. Chemokines were key mediators of cell migration during routine immune surveillance, inflammation and development. 2. Many chemokines and their receptors can be effective therapeutic targets for MS. | 106,107 |
| COMPLEMENT AND COAGULATION CASCADES | 32 | Complement and coagulation were major blood-borne proteolytic cascades. | 73 |
| T CELL RECEPTOR SIGNALING PATHWAY | 27 | T-cell receptors(TCRs) were indispensable elements of the immune system. | 108 |
| MAPK SIGNALING PATHWAY | 26 | Critical for cell survival and proliferation, cell adhesion and chemotaxis, and pro-inflammatory responses of immune cells. | 75 |
| CELL ADHESION MOLECULES CAMS | 23 | 1. In the immune system, CAMs regulated cell development, activation, differentiation, migration and many other cellular processes of crucial importance for the immune response. 2. Involved in the regulation of synaptic plasticity and the formation of neuronal networks. | 79-81 |
| NEUROACTIVE LIGAND RECEPTOR INTERACTION | 23 | 1)Closely related to neurological function  2)Impact on memory capacity. | 78 |
| CYTOSOLIC DNA SENSING PATHWAY | 18 | Cytosolic DNA sensors expressed by resident CNS cells recognized endogenous DNA and triggered immune responses in peripheral cell types. | 109 |

**Table S6** The top 10 KEGG pathways with minimum FDR containing inflammatory markers

| FDR | KEGG |
| --- | --- |
| 0.001626 | NICOTINATE AND NICOTINAMIDE METABOLISM |
| 0.012239 | HEMATOPOIETIC CELL LINEAGE |
| 0.012239 | HEMATOPOIETIC CELL LINEAGE |
| 0.078208 | B CELL RECEPTOR SIGNALING PATHWAY |
| 0.078208 | B CELL RECEPTOR SIGNALING PATHWAY |
| 0.078208 | B CELL RECEPTOR SIGNALING PATHWAY |
| 0.078208 | B CELL RECEPTOR SIGNALING PATHWAY |
| 0.078208 | B CELL RECEPTOR SIGNALING PATHWAY |
| 0.078208 | B CELL RECEPTOR SIGNALING PATHWAY |
| 0.078208 | B CELL RECEPTOR SIGNALING PATHWAY |

**Table S7** Top 10 most enriched BP terms containing inflammatory markers

| BP | Enriched shortest paths | Functions | Reference |
| --- | --- | --- | --- |
| DEFENSE RESPONSE(GO:0006952) | 88 | The defense response was a self-protective reaction of the organism. | 112 |
| NEGATIVE REGULATION OF DEFENSE RESPONSE(GO:0031348) | 80 | it adversely affected the human health and inflammation was a defense response, excessive inflammation may cause damage to normal tissue cells and aggravate MS. | 113 |
| NEGATIVE REGULATION OF CYTOKINE PRODUCTION(GO:0001818) | 76 | Cytokines were powerful and critical mediators of normal immune system function and negative regulation could negatively impact immunity. | 114 |
| LYMPHOCYTE MEDIATED IMMUNITY(GO:0002449) | 73 | Immune mechanisms associated with aberrant lymphocyte activity were a major cause of neuronal damage, which were likely to be responsible for the pathogenesis of MS. | 115 |
| REGULATION OF LEUKOCYTE MEDIATED IMMUNITY(GO:0002703) | 71 | Leukocyte infiltration into the CNS was an important step in the neuropathogenesis of MS. | 116 |
| NEGATIVE REGULATION OF VIRAL INDUCED CYTOPLASMIC PATTERN RECOGNITION RECEPTOR SIGNALING PATHWAY(GO:0039532) | 71 | Pattern recognition receptors(PRRs) that recognized microorganisms were the first line of defense against potential pathogens. | 117 |
| HUMORAL IMMUNE RESPONSE(GO:0006959) | 69 | The humoral immune system plays a role in the initiation and regulation of the inflammatory response. | 100 |
| REGULATION OF LEUKOCYTE PROLIFERATION(GO:0070663) | 69 | Leukocyte infiltration into the CNS was an important step in the neuropathogenesis of MS. | 116 |
| NEGATIVE REGULATION OF INFLAMMATORY RESPONSE(GO:0050728) | 68 | Inflammatory response les to demyelination and early neuronal transection. | 118 |
| POSITIVE REGULATION OF INFLAMMATORY RESPONSE(GO:0050729) | 65 | Inflammatory response led to demyelination and early neuronal transection. | 118 |

**Table S8** The top ten BP terms with minimum FDR containing inflammatory markers

| FDR | BP |
| --- | --- |
| 0.001638 | MACROPHAGE COLONY STIMULATING FACTOR SIGNALING PATHWAY(GO:0038145) |
| 0.001638 | REGULATION OF CELLULAR RESPONSE TO MACROPHAGE COLONY STIMULATING FACTOR STIMULUS(GO:1903972) |
| 0.001638 | MICROGLIAL CELL MIGRATION(GO:1904124) |
| 0.002896 | CELLULAR RESPONSE TO MACROPHAGE COLONY STIMULATING FACTOR STIMULUS(GO:0036006) |
| 0.003687 | POSITIVE REGULATION OF MICROGLIAL CELL ACTIVATION(GO:1903980) |
| 0.003978 | RESPONSE TO MACROPHAGE COLONY STIMULATING FACTOR(GO:0036005) |
| 0.003978 | REGULATION OF GLIAL CELL MIGRATION(GO:1903975) |
| 0.007955 | POSITIVE REGULATION OF NEUROINFLAMMATORY RESPONSE(GO:0150078) |
| 0.007955 | REGULATION OF MICROGLIAL CELL ACTIVATION(GO:1903978) |
| 0.009472 | REGULATION OF MACROPHAGE CHEMOTAXIS(GO:0010758) |

**Table S9** KEGG pathways in each cancer with minimum FDR

| Type of cancer | FDR | KEGG | Functions | Reference |
| --- | --- | --- | --- | --- |
| BLCA | 0.0781 | RIBOFLAVIN METABOLISM | There was a negative correlation between riboflavin and cancer. | 127 |
| BRCA | 0.0820 | ALPHA LINOLENIC ACID METABOLISM | Dietary intake of ALA was associated with a slightly higher risk of cancer death. | 128 |
| COAD | 0.0605 | ASCORBATE AND ALDARATE METABOLISM | 1. ascorbate was used to disrupt tumor cell redox homeostasis, which in turn promotes cancer cell death, and might be a treatment for cancer. 2. The pathway related to cancer | 129,130 |
| ESCA | 0.0654 | TAURINE AND HYPOTAURINE METABOLISM | Taurine had anti-tumor properties in cancer | 131 |
| HNSC | 0.0767 | RIBOFLAVIN METABOLISM | There was a negative correlation between riboflavin and cancer. | 127 |
| KICH | 0.0630 | ASCORBATE AND ALDARATE METABOLISM | ascorbate was used to disrupt tumor cell redox homeostasis, which in turn promotes cancer cell death, and might be a treatment for cancer.  The pathway related to cancer | 129,130 |
| KIRC | 0.0655 | ASCORBATE AND ALDARATE METABOLISM | ascorbate was used to disrupt tumor cell redox homeostasis, which in turn promotes cancer cell death, and might be a treatment for cancer.  The pathway related to cancer | 129,130 |
| KIRP | 0.0799 | TAURINE AND HYPOTAURINE METABOLISM | Taurine had anti-tumor properties in cancer | 131 |
| LIHC | 0.0808 | TAURINE AND HYPOTAURINE METABOLISM | Taurine had anti-tumor properties in cancer | 131 |
| LUAD | 0.0545 | ASCORBATE AND ALDARATE METABOLISM | ascorbate was used to disrupt tumor cell redox homeostasis, which in turn promotes cancer cell death, and might be a treatment for cancer.  The pathway related to cancer | 129,130 |
| LUSC | 0.0614 | ASCORBATE AND ALDARATE METABOLISM | ascorbate was used to disrupt tumor cell redox homeostasis, which in turn promotes cancer cell death, and might be a treatment for cancer.  The pathway related to cancer | 129,130 |
| PRAD | 0.0540 | ASCORBATE AND ALDARATE METABOLISM | ascorbate was used to disrupt tumor cell redox homeostasis, which in turn promotes cancer cell death, and might be a treatment for cancer.  The pathway related to cancer | 129,130 |
| READ | 0.0617 | ASCORBATE AND ALDARATE METABOLISM | ascorbate was used to disrupt tumor cell redox homeostasis, which in turn promotes cancer cell death, and might be a treatment for cancer.  The pathway related to cancer | 129,130 |
| STAD | 0.0654 | TAURINE AND HYPOTAURINE METABOLISM | Taurine had anti-tumor properties in cancer | 131 |
| THCA | 0.0403 | ASCORBATE AND ALDARATE METABOLISM | ascorbate was used to disrupt tumor cell redox homeostasis, which in turn promotes cancer cell death, and might be a treatment for cancer.  The pathway related to cancer | 129,130 |
| UCEC | 0.0623 | ASCORBATE AND ALDARATE METABOLISM | ascorbate was used to disrupt tumor cell redox homeostasis, which in turn promotes cancer cell death, and might be a treatment for cancer.  The pathway related to cancer | 129,130 |

**Table S10** BP terms in each cancer with minimum FDR

| Type of cancer | FDR | BP | Functions | Reference |
| --- | --- | --- | --- | --- |
| BLCA | 0.0455 | REGULATION OF PHENOTYPIC SWITCHING(GO:1900239) | As a target for cancer therapy. | 132 |
| BRCA | 0.0415 | SYNAPTIC SIGNALING VIA NEUROPEPTIDE(GO:0099538) | Neural mechanisms were inverted in cancer to promote malignant cancer growth. | 133 |
| COAD | 0.0603 | FLAVONOID GLUCURONIDATION(GO:0052696) | Flavonoid metabolism posed a significant risk for chemotherapy-induced peripheral neuropathy(CIPN) in cancer patients. | 134 |
| COAD | 0.0603 | XENOBIOTIC GLUCURONIDATION(GO:0052697) | Flavonoid metabolism posed a significant risk for chemotherapy-induced peripheral neuropathy(CIPN) in cancer patients. | 134 |
| COAD | 0.0603 | SPERM MITOCHONDRIAL SHEATH ASSEMBLY(GO:0120317) |  |  |
| COAD | 0.0603 | NEGATIVE REGULATION OF MYOBLAST PROLIFERATION(GO:2000818) | Associated with muscle wasting, which was a key feature of cancer-related cachexia. | 135 |
| ESCA | 0.0701 | PROTEIN LOCALIZATION TO CENP A CONTAINING CHROMATIN(GO:0061644) | Overexpression and mislocalization of CENP-A could be observed in cancer, which was associated with increased cancer aggressiveness. | 136 |
| HNSC | 0.0282 | NEGATIVE REGULATION OF MYOBLAST PROLIFERATION(GO:2000818) | Associated with muscle wasting, which was a key feature of cancer-related cachexia. | 135 |
| KICH | 0.0683 | SYNAPTIC SIGNALING VIA NEUROPEPTIDE(GO:0099538) | Neural mechanisms were inverted in cancer to promote malignant cancer growth. | 133 |
| KIRC | 0.0538 | PROTEIN LOCALIZATION TO CENP A CONTAINING CHROMATIN(GO:0061644) | Overexpression and mislocalization of CENP-A could be observed in cancer, which was associated with increased cancer aggressiveness. | 136 |
| KIRC | 0.0538 | NEGATIVE REGULATION OF CELL CHEMOTAXIS TO FIBROBLAST GROWTH FACTOR(GO:1904848) | Had carcinogenic potential, but can be tumor suppressor in some cases. | 137 |
| KIRP | 0.0444 | PROTEIN LOCALIZATION TO CENP A CONTAINING CHROMATIN(GO:0061644) | Overexpression and mislocalization of CENP-A could be observed in cancer, which was associated with increased cancer aggressiveness. | 136 |
| LIHC | 0.0300 | PROTEIN LOCALIZATION TO CENP A CONTAINING CHROMATIN(GO:0061644) | Overexpression and mislocalization of CENP-A could be observed in cancer, which was associated with increased cancer aggressiveness. | 136 |
| LUAD | 0.0359 | FLAVONE METABOLIC PROCESS(GO:0051552) | Playing a role in cancer prevention and treatment. | 138 |
| LUAD | 0.0359 | PROTEIN LOCALIZATION TO CENP A CONTAINING CHROMATIN(GO:0061644) | Overexpression and mislocalization of CENP-A could be observed in cancer, which was associated with increased cancer aggressiveness. | 136 |
| LUSC | 0.0703 | PROTEIN LOCALIZATION TO CENP A CONTAINING CHROMATIN(GO:0061644) | Overexpression and mislocalization of CENP-A could be observed in cancer, which was associated with increased cancer aggressiveness. | 136 |
| PRAD | 0.0240 | GONADAL MESODERM DEVELOPMENT(GO:0007506) | Intermediate mesoderm derived urogenital system. | 139 |
| READ | 0.0660 | SPERM MITOCHONDRIAL SHEATH ASSEMBLY(GO:0120317) |  |  |
| STAD | 0.0602 | RNA 3 URIDYLATION(GO:0071076) | As a critical gene regulator and driver of tumorigenesis. | 140 |
| THCA | 0.0262 | XENOBIOTIC GLUCURONIDATION(GO:0052696) | Flavonoid metabolism posed a significant risk for chemotherapy-induced peripheral neuropathy(CIPN) in cancer patients. | 134 |
| UCEC | 0.0614 | PROTEIN LOCALIZATION TO CENP A CONTAINING CHROMATIN(GO:0061644) | Overexpression and mislocalization of CENP-A could be observed in cancer, which was associated with increased cancer aggressiveness. | 136 |
| UCEC | 0.0614 | SPERM MITOCHONDRIAL SHEATH ASSEMBLY(GO:0120317) |  |  |
| UCEC | 0.0614 | NEGATIVE REGULATION OF MYOBLAST PROLIFERATION(GO:2000818) | Associated with muscle wasting, which was a key feature of cancer-related cachexia. | 135 |
