# Supplementary figures and images for: Identification of crucial inflammaging related risk factors in multiple sclerosis
Source: Front Mol Neurosci. 2024 May 21;17:1398665. doi: 10.3389/fnmol.2024.1398665 (PMC11148336; doi:10.3389/fnmol.2024.1398665)

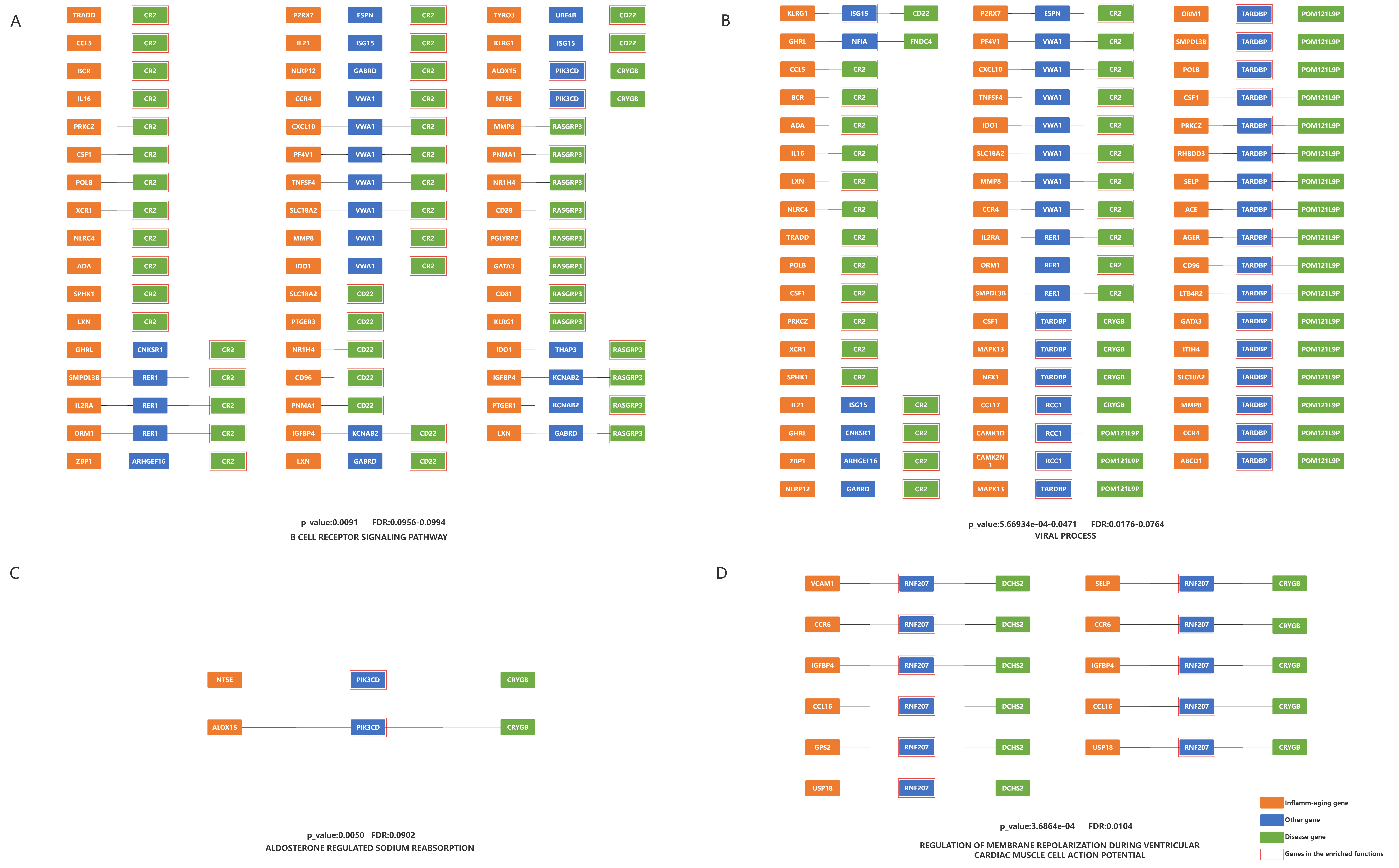

Supplement: Supplementary Figure S1 — Enrichment analysis of the shortest paths of KEGG and BP, before combining overlap shortest paths. (A) KEGG with the most shortest enriched paths; (B) BP with the most shortest enriched paths; (C) KEGG with the minimum FDR; (D) BP with the minimum FDR. The orange nodes represent the inflammaging markers, the blue nodes represent the genes connecting inflammaging markers and disease markers, the green nodes represent the disease markers, and the genes in the red square frames coincide with those genes in the enriched functions. [file Data_Sheet_1.ZIP › Supplemental files/Figure S1.tif]

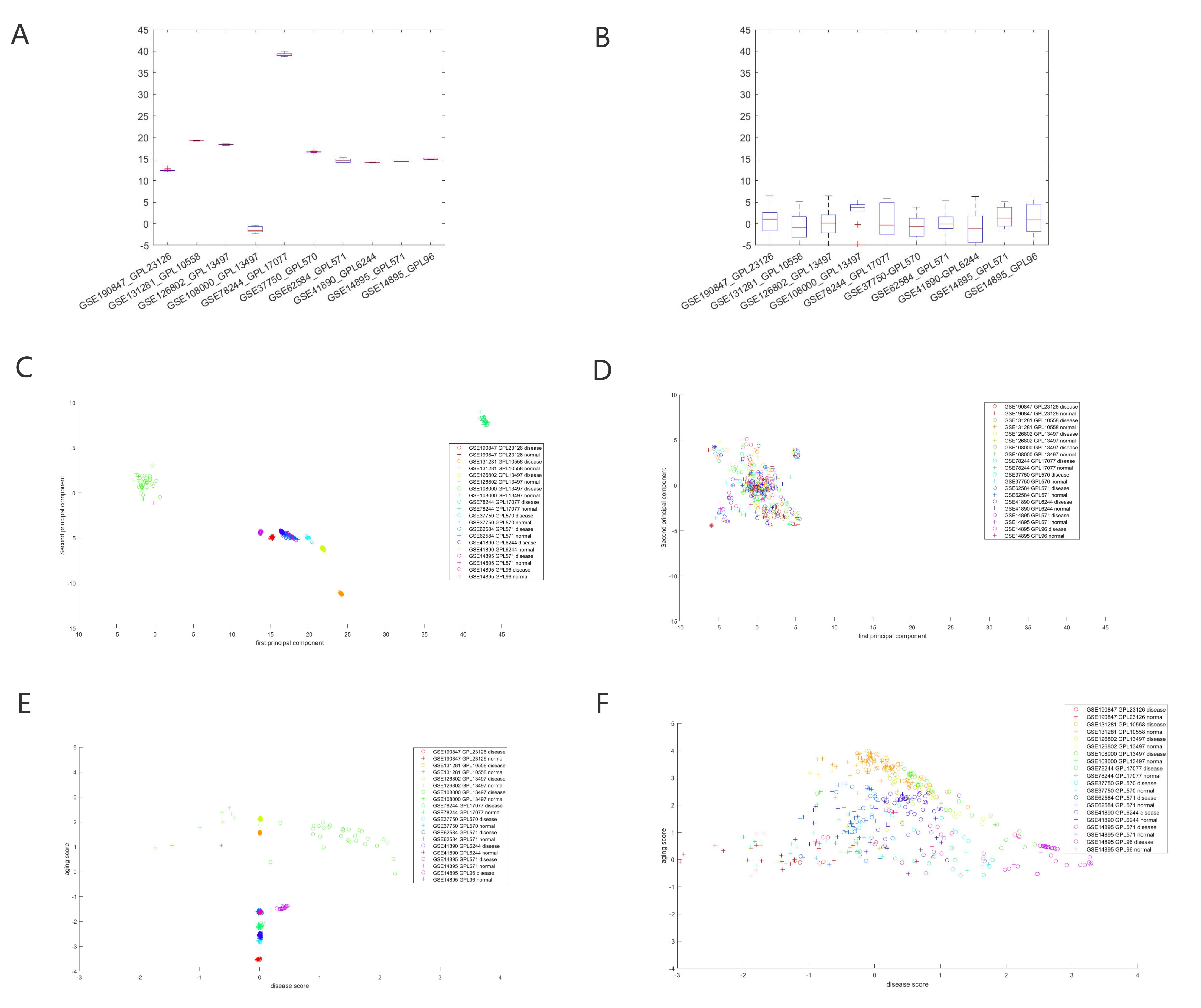

Supplement: Supplementary Figure S1 — Enrichment analysis of the shortest paths of KEGG and BP, before combining overlap shortest paths. (A) KEGG with the most shortest enriched paths; (B) BP with the most shortest enriched paths; (C) KEGG with the minimum FDR; (D) BP with the minimum FDR. The orange nodes represent the inflammaging markers, the blue nodes represent the genes connecting inflammaging markers and disease markers, the green nodes represent the disease markers, and the genes in the red square frames coincide with those genes in the enriched functions. [file Data_Sheet_1.ZIP › Supplemental files/Figure S2.tif]
